# Impacts of Behavioral Compliance on Weight-Loss and Metabolic Profile in a Smartphone App-Based Lifestyle Intervention or Plus Dietitian Supporting: A Randomized and Controlled Trial Among Chinese

**DOI:** 10.1007/s43657-024-00162-0

**Published:** 2025-01-30

**Authors:** Xue Li, Ling Lu, Liang Sun, Yunxia Xie, Kang Huang, Changzheng Yuan, Liying Chen, Xu Lin

**Affiliations:** 1 Key Laboratory of Systems Health Science of Zhejiang Province, School of Life Science, Hangzhou Institute for Advanced Study, University of Chinese Academy of Sciences, Chinese Academy of Sciences, Hangzhou, 310024 China; 2 Ministry of Education Key Laboratory of Public Health Safety, School of Public Health, Institute of Nutrition, Fudan University, Shanghai, 200032 China; 3 Nursing Department, Sir Run Run Shaw Hospital, Zhejiang University School of Medicine, Hangzhou, 310016 China; 4 School of Public Health, the Second Affiliated Hospital, Zhejiang University School of Medicine, Hangzhou, 310058 China; 5 Department of Nutrition, Harvard T.H. Chan School of Public Health, Boston, 02115 USA; 6 Department of General Practice, Sir Run Run Shaw Hospital, School of Medicine, Zhejiang University, Hangzhou, 310016 China; 7 Shanghai Institute of Nutrition and Health, University of Chinese Academy of Sciences, Chinese Academy of Sciences, 320 Yue-yang Rd., Shanghai, 200031 China

**Keywords:** Behavioral phenotypes, Compliance, Smartphone app-based lifestyle intervention, Weight loss, Randomized controlled trial

## Abstract

Mobile health technologies provided innovative solutions for lifestyle interventions and offered reliable methods to evaluate behavioral phenotypes during such interventions. To systematically quantify the impacts of behavioral compliance on weight-loss and metabolic profiles during lifestyle intervention, a total of 395 Chinese adults with overweight/obesity (BMI ≥ 24 kg/m^2^) or central obesity (waistline ≥ 90 cm for men or ≥ 80 cm for women) were randomly assigned to a smartphone app-based arm (SAA, n = 197) or smartphone app plus dietitian arm (SADA, n = 198) for 6 months. Compliance scores (0–5) were determined based on fulfilling five behavioral tasks: completing online courses, wearing a smart band, and recording weight, food intake, and blood pressure. SADA had greater weight-loss (− 4.94% vs. − 2.28%, *p* < 0.001) and lower triglyceride, but higher HDL-C levels (both *p* < 0.05) than SAA after six months. Between-group weight-loss differences were attenuated at compliance scores ≥ 3 (SADA: − 6.30% vs. SAA: − 4.79%, *p* = 0.07). Mediation analysis suggested that compliance scores explained approximately 30% of the additional weight loss in the SADA (*p* < 0.001), and self-weighing was the primary mediator (*p* < 0.05). Higher educational levels, greater initial weight loss, self-perceived simplicity, and satisfaction with the program were potential determinants of intervention compliance. Overall, the superior weight loss and metabolic improvements in the SADA group could be mediated by behavioral compliance, which was possibly influenced by some demographic and intervention response features. Our findings highlighted the roles of behavioral phenotypes and adherence in app-based lifestyle interventions, and added evidence for the future development of more precise strategies in long-term weight management.

**Supplementary Information:**

The online version contains supplementary material available at 10.1007/s43657-024-00162-0.

## Introduction

The prevalence of obesity has tripled in the last few decades, affecting approximately two billion adults worldwide (Haththotuwa et al. 2020). Approximately 85 million or more than 50% of Chinese adults are overweight or obese (Abarca-Gómez et al. 2017; Wang et al. 2021). The accumulation of excess body fat, particularly abdominal adipose tissue, is more prevalent in Asians and plays a vital role in the pathogenesis of cardiometabolic diseases such as metabolic syndrome (MetS), type 2 diabetes, cardiovascular diseases, and certain cancers (Bhaskaran et al. 2014; Ghaben et al. 2019; Powell-Wiley et al. 2021). Compelling evidence has demonstrated that lifestyle (diet and exercise) modification is the safest and most adopted strategy for weight management in more than half a century (Bray et al. 2016). However, traditional face-to-face health education or dietary feeding trials require tremendous manpower and costs, which hence are not practical in routine primary care (Jensen et al. 2014; Pagoto 2011; Wadden et al. 2020). Moreover, due to the relapsing nature, preventing weight regain following weight loss remains a lifelong battle for obese people (Bray et al. 2017). Therefore, an effective and affordable intervention strategy is urgently needed to address the current obesity epidemic.

With rapidly emerging new health technologies in the last few decades, app-based or other portable device-based lifestyle interventions have attracted increasing attention to improving record accuracy in the management of obesity and type 2 diabetes (Association 2020; Chew et al. 2022; Serrano et al. 2016). These widely accessible and cost-effective intervention strategies provide a promising alternative for personalized weight management. It was estimated that 84% of the population in Asian countries will have smartphones by 2025, and over 1.04 billion Chinese people had access to the internet via their mobile phones by 2022 (50th China Statistical Report on Internet Development 2022; The mobile economy Asia Pacific 2020). However, existing studies have yielded inconsistent results, and app-based interventions on weight loss without dietitian coaching were less effective (ranging from 0.12 to 4.32 kg) than those with dietitian coaching (ranging from 1.57 to 5.11 kg) (Beleigoli et al. 2020; Chang et al. 2022). Therefore, it is critically important to identify major factors that affect the effectiveness of potential eHealth applications in community-based large-scale overweight/obese and high-risk populations.

Adjusting behavioral phenotypes according to the lifestyle intervention instructions is crucial for successful weight loss and metabolic improvement. Integrating app-based and wearable technology into a weight-loss program could accurately record behavioral phenotypes and assess compliance with the intervention instructions by improving the reliability of participants’ records and alleviating their self-tracking burden. However, most previous lifestyle intervention studies still adopted a self-report design, and compliance was evaluated by heterogeneous measurements across studies (Chopra et al. 2021). Therefore, it is unclear which factor(s) or to what extent these factor(s) could influence participants’ adherence to lifestyle interventions, as well as health outcomes. Moreover, compliance with lifestyle interventions may vary across populations (Ang et al. 2021). To date, most smartphone app-based weight-loss trials have been conducted in Western populations, and few high-quality randomized control trials (RCTs) have been performed in Asian populations, who have varied genetic backgrounds, habitual diets and lifestyles, obese phenotypes, and disease predispositions compared with Western populations (Fan et al. 2017). In other words, new insights into the heterogeneity of behavioral compliance are needed to develop more targeted prevention and treatment approaches tailored to individual predisposition.

Therefore, in this 6-month RCT, we integrated a smartphone app-based lifestyle intervention with or without dietitian support, as well as scored behavioral compliance based on directly measured participants’ fulfillment of five required tasks, such as completing app-based online courses learning, recording food intake, and monitoring changes in weight, steps, and blood pressure by multiple app-connected wireless devices, this study aimed to investigate: (1) differences in the efficacy of a smartphone app-based arm (SAA) and a smartphone app plus dietitian arm (SADA) for weight loss and metabolic improvement; (2) to what extent between-group differences can be explained by behavioral compliance (five tasks collectively or individually); and (3) what factors and how these factors influence the compliance levels among 395 Chinese individuals with overweight/obesity and high metabolic risks.

## Materials and Methods

### Study Design

This 6-month single-blinded, parallel, RCT was conducted from March 2021 to November 2021. The study protocol was approved by the institutional review boards of the Shanghai Institute of Nutrition and Health, the Chinese Academy of Sciences (ER-SINH-252004), and Sir Run Run Shaw Hospital, Zhejiang University School of Medicine (20200923-11). All participants provided written informed consent before participating in the study.

### Participants

**Fig. 1:**
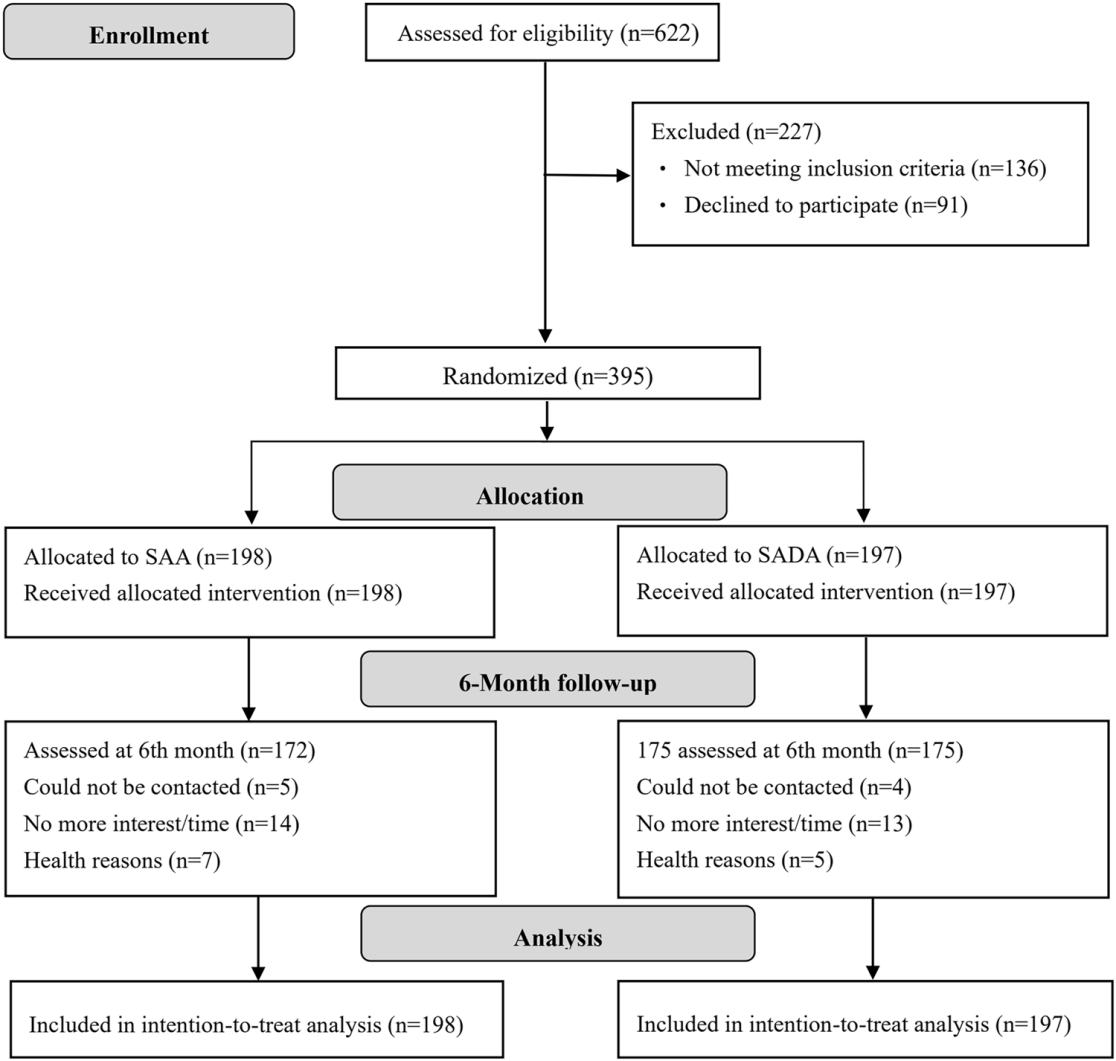
Study flow diagram. Abbreviations: SAA, Smartphone app-based arm; SADA, Smartphone app-based plus dietitian arm

Participants were recruited among faculty and staff members from three universities located in Hangzhou, Zhejiang Province, China, by advertisements. After prescreening with an annual health examination and completion of a short questionnaire administered by the research team, 622 potentially eligible participants were invited to participate in a screening visit at Sir Run Run Hospital. The eligibility criteria were as follows: (1) individuals aged 20–65 years; (2) those who were overweight/obese [Body mass index (BMI) ≥ 24 kg/m^2^, defined by the Chinese criteria (Pan et al. 2021)] or centrally obese (defined as a waist circumference ≥ 90 cm for men or ≥ 80 cm for women); and (3) those who had access to and were able to use a smartphone. The following individuals were excluded: individuals with uncontrolled diabetes, dyslipidemia, or hypertension, severe cardiovascular, liver, or kidney disease, or cancer; individuals with psychological disorders, heavy alcohol use, or infectious diseases; women who were pregnant or lactating; and individuals who had participated in other studies within three months before the current study (Supplemental Methods). After the screening, 136 individuals did not meet the inclusion criteria, and 91 individuals declined to participate (Fig. 1).

### Randomization and Masking

Eligible participants were randomly assigned (1:1) into one of the two groups, and an independent statistician, who was unaware of the treatment allocation, performed the randomization with stratification by sex, medians of age, body weight, systolic blood pressure, fasting plasma glucose, and triglyceride levels using Statistical Analysis System (SAS) software (version 9.4). The allocation sequence was concealed from the participants until they completed the baseline visit. The researchers who assessed the study outcomes and analyzed the data were blinded to the group assignment.

### Interventions

The basic intervention program for both the SADA and SAA groups included smartphone app-based online healthy lifestyle courses and dietary recommendations combined with multiple app-based monitoring devices. Before undergoing the baseline assessment, all eligible participants obtained access to an interactive smartphone app (Nutriease, Zhejiang, China) and Bluetooth devices, including an electronic sphygmomanometer (YE690AR, Yuwell), a digital weighing scale (NT-2016C, Nutriease, Zhejiang, China), and a smart band (CW10C, Nutriease, Zhejiang, China). These devices enabled the wireless transfer of data to both the participants’ smartphone apps (Nutriease, Zhejiang, China) and cloud servers in real-time. During the 6-month intervention, participants in both groups were asked to measure their body weight and blood pressure three times per week and to wear the smart band to monitor their daily steps.

In the initial 98 days, the app-based health lifestyle courses, which were adapted from the Diabetes Prevention Program (DPP) with some modifications, were provided daily to all participants in both groups (“The Diabetes Prevention Program (DPP): description of lifestyle intervention” 2002; Group 2002). The course contents included instructions on setting weight-loss goals, modifying the diet for healthy eating, encouraging physical activity, and providing psychosocial and behavioral guidance to overcome potential barriers, enhance motivation, and prevent confidence collapse during the weight-loss process.

To achieve weight reduction, participants in both groups received a standardized phone call from a dietitian at baseline to obtain dietary recommendations and health lifestyle instructions. Participants were asked to reduce their caloric intake from their pretrial energy requirement by 30%, which was estimated as the baseline body weight  × 25 kcal/kg × 70% (Jensen et al. 2014; Zhu 2021). Calorie intake from carbohydrates, protein, and fat accounted for 40%, 30%, and 30% of the diet, respectively. In principle, all participants received standard recipes and dietary recommendations to consume less refined carbohydrates and saturated fat, along with more vegetables, fruits, and high-quality protein. Participants with elevated glucose and blood pressure levels were additionally reminded to avoid high glycemic index (GI) foods and reduce salt intake in their daily diets, respectively. After participants completed the 6-month intervention or when they reached the target of 15% weight loss or a BMI equivalent to 21 kg/m^2^ during the intervention, they received a dietary recommendation to meet the 100% estimated energy requirement for maintaining their weight. To track dietary intake, the participants were encouraged to record their meals via the app as frequently as possible.

In addition to the aforementioned basic intervention program, participants in the SADA group received intensive online coaching from a dietitian, who was responsible for reviewing trial progress, addressing inquiries, and encouraging the participants to follow the instructions. The role of the dietitian was to promote participant engagement with the intervention program and the adoption of a healthy lifestyle. The dietitian tracked the participants’ records of self-monitoring items and their engagement with education sessions via a web-based platform. The participants in the SADA group received at least three phone calls from the dietitian in the first week of the intervention then weekly calls for the remaining time of the 6-month trial. These standardized outreaches were conducted according to structured forms and recorded in the web-based platform.

### Outcomes and Measurements

The primary outcome was the difference in the change in body weight (kg and percentage of initial body weight) at the end of the 6-month intervention between the SAA and SADA groups, while the secondary outcomes included the between-group changes in obesity-related measurements (BMI, waist circumference, body fat mass and lean mass percentages, and visceral fat area), metabolic profiles (fasting plasma glucose, insulin, blood pressure, total cholesterol, triglyceride, low-density lipoprotein cholesterol (LDL-C) and high-density lipoprotein cholesterol (HDL-C) levels), effects of compliance with the lifestyle intervention, and major factors that affected compliance. In post hoc analyses, the reversion rates of MetS and its components were compared between the SAA and SADA groups.

MetS was defined by the updated National Cholesterol Education Program Adult Treatment Panel III (NCEP ATPIII) criteria for Asians (Supplemental Methods) (Alberti et al. 2009). The reversion rates of MetS and its components were defined as the proportion of individuals that met the criteria for MetS or its components at baseline but not after the 6-month intervention.

At baseline and the end of the 6-month intervention, all participants were invited to undergo physical examinations at Sir Run Run Shaw Hospital. Questionnaires were completed and anthropometric measurements and biological samples were collected following a standard protocol (Wu et al. 2010). At baseline, a face-to-face interview was conducted by the research team to obtain information on demographic characteristics, lifestyle factors, health status, and the use of medications and nutritional supplements (Ye et al. 2007). During each visit, physical activity levels were evaluated by the short form of the International Physical Activity Questionnaire (Craig et al. 2003). A 3-day dietary record was completed online by dietitians. The Adherence to a Healthy Lifestyle Questionnaire (AHLQ) was used to evaluate self-perceived motivation, barriers, simplicity, and satisfaction with the program at the end of the 6-month intervention (Malekzadeh et al. 2022). Body composition was assessed via a bioelectrical impedance device (InBody 720). Weight, height, waist circumference, and blood pressure were measured following a previously described method, and BMI was calculated as weight (kg)/height (m^2^) (Luo et al. 2022). At baseline and 6-month, overnight fasting (10 h) blood samples were collected in tubes containing liquid ethylenediaminetetra-acetic acid (EDTA), centrifuged immediately at 4 °C, aliquoted, and then stored at − 80 °C until analysis. Plasma concentrations of glucose, triglycerides, total cholesterol, HDL-C, and LDL-C were measured on automatic biochemical analyzers (LDL-C: Roche Cobas C701; other biomarkers: Roche Cobas C502) using commercial reagents from Maccura Biotechnology Co., Ltd. All assessors were blinded to group allocation.

Compliance with the intervention was defined as the extent to which participants fulfilled the five required core tasks, namely, completing online learning courses, wearing the smart band, and self-monitoring body weight, blood pressure, and dietary intake (e.g., monitoring body weight compliance = [the number of weeks the recommended monitoring frequency was fulfilled]/24 weeks). Participants were considered compliant in each core task if their adherence to that behavior ranked in the upper half among all participants, and a score of one point was given; otherwise, zero points were assigned. A compliance score for the overall compliance was generated by summing the individual scores for the five different behaviors (ranging 0–5). Because of the generally low frequency of blood pressure monitoring, in the calculation of the compliance score, one point was assigned when participants measured their blood pressure once per week instead of three times per week. The overall compliance score ranged from zero to five, with a higher score indicating better implementation of the entire intervention program.

### Statistical Analysis

For the primary outcome of body weight, approximately 390 eligible participants (195 per group) were needed to achieve a significant between-group difference in weight loss > 2 kg after the 6-month intervention when considering a standard deviation (SD) of 6 kg, a power of 90%, a 2-sided significance level of 5%, and a dropout rate of 20% (Thomas et al. 2015). Power Analysis & Sample Size (PASS) software (version 13.0.6) was used for the sample size calculation.

The analyses followed the intent-to-treat (ITT) principle. Multiple imputations were performed for missing values with the mice package in R using random forest imputations and pooling estimation from five imputed datasets and 50 iterations with both predictors and outcomes, as well as with other variables regarded as important to explain the missing values (van Buuren et al. 2011). Variables with skewed distributions were log-transformed before analysis. Data are presented as the mean (SD) or median (interquartile range, IQR) for continuous variables with a normal or skewed distribution and n (%) for categorical variables. Baseline characteristics between groups were compared using independent *t-*tests, Wilcoxon tests, or χ^2^ tests when appropriate to determine the baseline comparability and check randomization.

The effects of the intervention group, time, and group-by-time interactions on continuous outcomes were examined via the linear mixed effects model with the covariates of age and sex as the fixed effects and individual as the random (intercept) effect, assuming an unstructured variance–covariance matrix. Poisson regression with a robust error variance was used to compare proportions of obtaining clinically significant weight loss (≥ 3%, ≥ 5%, or ≥ 10%) and the reversion rate of MetS and its components between treatment groups, adjusting for age and sex (Muramoto et al. 2014; Wing et al. 2011). Sensitivity analyses were conducted in per-protocol analysis to test the robustness.

The impact of participants’ compliance with the intervention program on weight loss was analyzed. Spearman correlations between compliance with each behavior and changes in obesity-related variables were assessed. Linear mixed effect models were performed to assess the group effect in compliant participants (defined as those with a compliance score ≥ 3) adjusting for age and sex. Mediation effects of compliance with the five required tasks (individually and collectively) on changes in body weight (kg, %) and BMI tracked weekly were evaluated with the mediation package in R (Tingley et al. 2014). Determinants associated with adherence were detected using multilevel logistic regression models with the MASS package in R (Venables et al. 2002). All analyses were conducted using R 4.1.2 assuming a two-tailed α of 0.05.

## Results

Among the 395 eligible participants, 347 (87.8%) completed the 6-month intervention (Fig. 1), while 48 (26 persons in the SAA group and 22 persons in the SADA group) dropped out due to a loss of contact or interest, a busy schedule, or an illness. Persons who dropped out had similar baseline characteristics as those who completed the 6-month trial (Table S1). No adverse events were reported during the six months.

### Baseline Characteristics

The average age of the participants was 43.6 ± 8.90 years, and 47.6% (188/395) of them were women. At baseline, 51.1% of the participants had MetS, 96.0% were overweight/obese, and 89.4% had central obesity. Participants in both groups showed comparable demographics and clinical characteristics (Table 1).

**Table 1:** Baseline characteristics of participants by intervention group

Characteristics	All (n = 395)	SAA (n = 198)	SADA (n = 197)	*p*-value
Age, year	43.6 (8.9)	43.3 (9.0)	43.9 (8.8)	0.48
Female, n (%)	188 (47.6)	94 (47.5)	94 (47.7)	1.00
Education ≥ 10 y, n. (%)	342 (86.6)	169 (85.4)	173 (87.8)	0.42
Current smoker, n. (%)^a^	50 (12.7)	27 (13.6)	23 (11.7)	0.68
Alcohol drinker, n. (%)^a^	237 (60.2)	121 (61.1)	116 (59.2)	0.77
Body weight, kg	75.2 (11.5)	74.9 (11.4)	75.6 (11.5)	0.53
BMI, kg/m^2^	27.7 (2.7)	27.5 (2.6)	27.8 (2.9)	0.34
Overweight/obesity, no. (%)	380 (96.2)	191 (96.5)	189 (95.9)	0.99
Body fat mass percentage, %	33.8 (6.3)	33.5 (6.2)	34.1 (6.3)	0.41
Body lean mass percentage, %	62.5 (6.0)	62.8 (5.9)	62.3 (6.1)	0.38
Visceral fat area, cm^2^	116.9 (21.4)	115.6 (21.3)	118.2 (21.4)	0.21
MetS, no. (%)	202 (51.1)	101 (51.0)	101 (51.3)	1.00
Elevated fasting glucose^a^	196 (49.8)	98 (49.8)	98 (49.8)	1.00
Low HDL-C	178 (45.1)	90 (45.5)	88 (44.7)	0.96
Elevated triglycerides^b^	145 (36.9)	69 (35.2)	76 (38.6)	0.56
Central obesity	353 (89.4)	174 (87.9)	179 (90.9)	0.42
Elevated blood pressure	135 (34.2)	65 (32.8)	70 (35.5)	0.65
≥ 2 MetS risk factors, no. (%)	310 (78.5)	152 (76.8)	158 (80.2)	0.48
Waist circumference, cm	93.5 (8.0)	93.0 (7.6)	93.9 (8.3)	0.24
Systolic blood pressure, mmHg	119.5 (14.8)	119.6 (14.8)	119.5 (14.8)	0.96
Diastolic blood pressure, mmHg	79.9 (9.8)	79.7 (9.5)	80.2 (10.2)	0.62
Fasting glucose, mmol/L^b^	5.6 (0.7)	5.6 (0.6)	5.6 (0.6)	0.85
Triglycerides mmol/L^c, d^	1.4 (1.1, 2.0)	1.4 (1.0, 2.0)	1.5 (1.1, 2.0)	0.33
Total cholesterol mmol/L	5.1 (1.0)	5.1 (1.0)	5.1 (1.0)	0.99
HDL-C mmol/L	1.2 (0.3)	1.2 (0.3)	1.2 (0.3)	0.87
LDL-C mmol/L	3.2 (0.9)	3.2 (0.9)	3.2 (0.9)	0.96

Values were presented as means (standard deviation) for continuous variables (unless indicated otherwise) and as numbers (%) for categorical variables. *p*-values were calculated using the student’s *t*-test and the χ^2^ test for continuous variables and categorical variables, respectively

HDL-C, high-density lipoprotein cholesterol; LDL-C, low-density lipoprotein cholesterol; MetS, metabolic syndrome; SAA, Smartphone app-based arm (control group); SADA, Smartphone app-based plus dietitian arm

^a^Information on smoking and drinking was missing for one participant in the SADA

^b^Glucose data was missing for one participant in the SAA owing to an extreme value (exceeding nine times IQR)

^c^Triglyceride data were missing for two participants in the SAA owing to extreme values (exceeding nine times IQR)

^d^Values were presented as median (interquartile range) due to the skewed distribution

### Changes in Body Weight and Metabolic Profiles

**Fig. 2:**
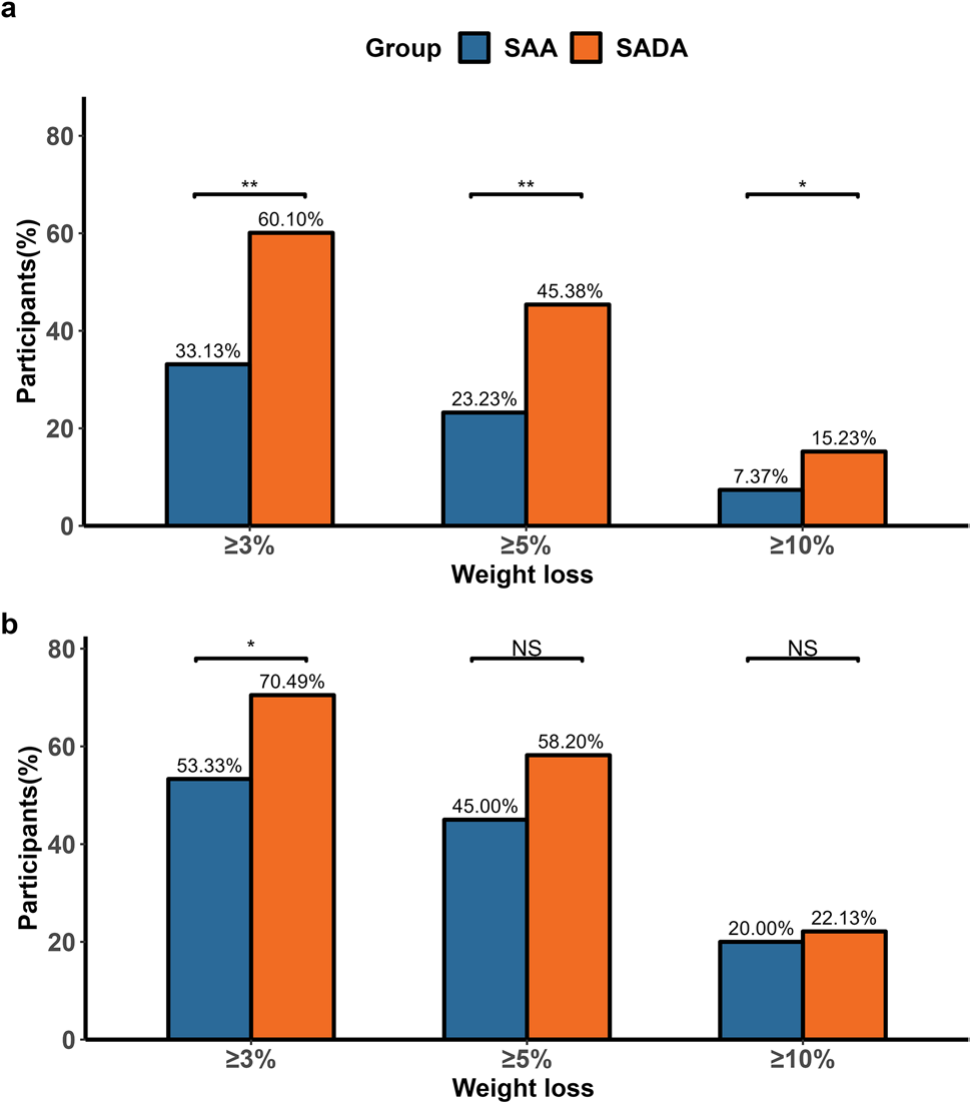
Effect of SADA compared to SAA on clinically significant weight change over 6-month intervention. The figure shows the percentage of participants with body weight loss of ≥ 3%, ≥ 5%, and ≥ 10% from baseline to the end of the 6-month intervention among (**a**) all participants allocated to SAA or SADA (SAA: n = 198; SADA: n = 197) and (**b**) participants who attended both baseline and 6-month follow-up with a compliance score ≥ 3. (SAA: n = 60; SADA: n = 122). Poisson regression with a robust error variance was used for between-group comparison adjusting for age and gender. *p-value < 0.05; **p-value < 0.01. Abbreviations: NS, not statistically significant; SAA, Smartphone app-based arm (control group); SADA, Smartphone app-based plus dietitian arm

**Fig. 3:**
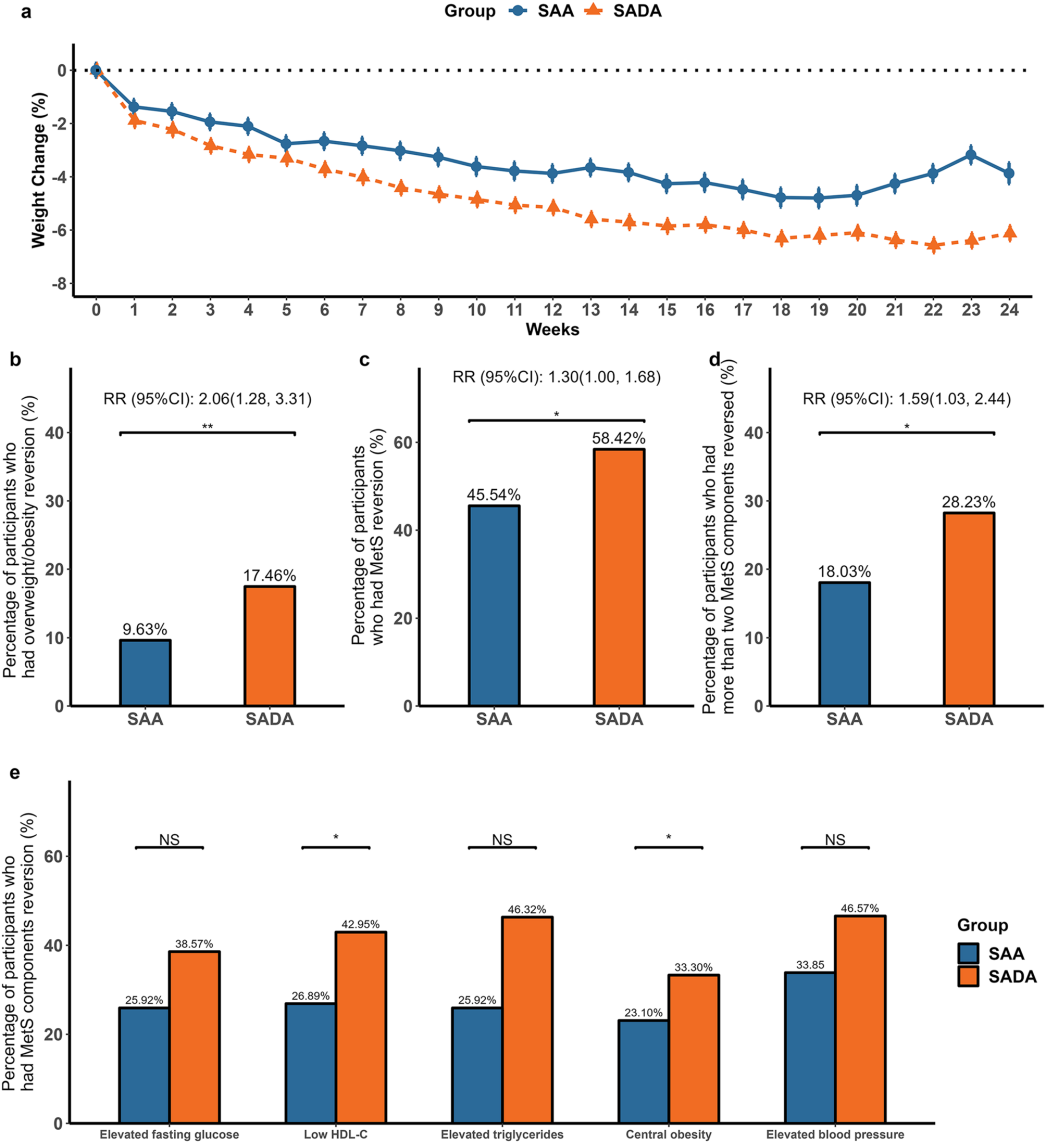
Effect of SADA compared to SAA on the trend of weight reduction and reversion rates of overweight, MetS, and MetS components over the 6-month intervention. Reversion of overweight/obesity, MetS, and MetS components was defined as participants with these conditions at baseline who no longer met the criteria for these conditions after the trial.** a** The figure shows the mean weight loss (%) monitored by blue-tooth scales over the 6-month intervention period, which were estimated by the linear mixed-effect models, among all participants allocated to SAA or SADA (SAA: n = 198; SADA: n = 197). **b** Overweight/obesity reversion rate at month six among overweight/obesity (BMI ≥ 24 kg/m^2^) participants at baseline (SAA: n = 191; SADA: n = 189). **c** MetS reversion rate at month six among participants with MetS at baseline (SAA: n = 101; SADA: n = 101). **d** The percentage of participants with more than two MetS components counts reversed at month six among participants with ≥ 2 MetS components at baseline (SAA: n = 152; SADA: n = 158). **e** Reversion rates of each MetS component at month six among participants with elevated fasting glucose (SAA: n = 98; SADA: n = 98), low HDL-C (SAA: n = 90; SADA: n = 88), elevated triglycerides (SAA: n = 69; SADA: n = 76), central obese (SAA: n = 174; SADA: n = 179), and elevated blood pressure (SAA: n = 65; SADA: n = 70) at baseline. *p-value < 0.05; **p-value < 0.01. Abbreviations: HDL-C, high-density lipoprotein cholesterol; MetS, metabolic syndrome; RR, relative rate; SAA, Smartphone app-based arm (control group); SADA, Smartphone app-based plus dietitian arm

As shown in Table 2, following a 6-month intervention, participants in the SADA group had greater weight loss than those in the SAA group (− 4.94% vs. − 2.28%, *p* = 0.005; − 3.59 kg vs. − 1.82 kg, *p* < 0.001, respectively). There was a significant interaction of group × time on BMI, percentage of fat mass, percentage of lean mass, and visceral fat area over the 6 months (all *p* < 0.01, Table 2, Model 1). The SADA was more likely to lead to clinically significant weight loss of ≥ 3%, ≥ 5%, and ≥ 10% after the 6-month intervention than the SAA (all *p* < 0.05) (Fig. 2a, Fig. S1). Figure 3a also demonstrates the trend of weight reduction over 6 months in both groups, with a more pronounced slope in the SADA group. The SADA group had a significantly higher relative rate (RR) of overweight/obesity reversion than the SAA group (*p* < 0.01) (Fig. 3b).

**Table 2:** Changes of anthropometrics and metabolic risk factors of participants from baseline to month six in the intention-to-treat analysis

	Mean change from baseline (SE)		Between-group difference model 1 (95% CI)	*p*-value	Between-group difference model 2 (95% CI)	*p*-value
	SAA (N = 198)	SADA (N = 197)				
Weight loss, kg	− 1.82 (0.47)*	− 3.59 (0.49)*	− 1.77 (− 3.00, − 0.55)	**0.005**	− 0.71 (− 1.97, 0.56)	0.276
Weight change, %	− 2.28 (0.38)*	− 4.94 (0.38)*	− 2.64 (− 3.64, − 1.64)	** < 0.001**	− 1.43 (− 2.46, − 0.41)	**0.007**
BMI, kg/m^2^	− 0.67 (0.15)*	− 1.34 (0.16)*	− 0.68 (− 1.12, − 0.23)	**0.004**	− 0.25 (− 0.70, 0.19)	0.270
Waist circumference, cm	− 3.64 (0.47)*	− 5.79 (0.50)*	− 2.15 (− 3.55, − 0.74)	**0.005**	− 0.76 (− 2.34, 0.83)	0.360
Systolic blood pressure, mmHg	− 2.15 (0.80)*	− 2.44 (0.79)*	− 0.29 (− 2.53, 1.94)	0.797	0.56 (− 1.85, 2.97)	0.651
Diastolic blood pressure, mmHg	− 0.91 (0.60)	− 2.24 (0.67)	− 1.33 (− 3.03, 0.36)	0.125	− 0.34 (− 2.12, 1.44)	0.709
Fasting glucose, mmol/L	0.02 (0.04)	− 0.04 (0.04)	− 0.07 (− 0.17, 0.04)	0.223	− 0.04 (− 0.15, 0.07)	0.567
Triglycerides mmol/L^a^	0.00 (0.03)	− 0.16 (0.04)*	− 0.16 (− 0.25, − 0.07)	**0.001**	− 0.10 (− 0.20, 0.00)	**0.046**
Total cholesterol mmol/L	0.11 (0.07)	0.08 (0.06)	− 0.03 (− 0.21, 0.14)	0.694	− 0.01 (− 0.19, 0.18)	0.964
HDL-C mmol/L	0.03 (0.02)	0.12 (0.02)*	0.08 (0.04, 0.13)	**0.001**	0.05 (0.00, 0.10)	**0.035**
LDL-C mmol/L	0.10 (0.06)	0.09 (0.06)	− 0.01 (− 0.16, 0.15)	0.921	0.00 (− 0.17, 0.17)	0.966
Body fat mass percentage, %	− 1.30 (0.33)*	− 2.82 (0.35)*	− 1.52 (− 2.49, − 0.55)	**0.004**	− 0.84 (− 1.86, 0.19)	0.118
Body lean mass percentage, %	1.32 (0.29)*	2.62 (0.29)*	1.30 (0.47, 2.14)	**0.003**	0.72 (− 0.14, 1.58)	0.101
Visceral fat area, cm^2^	− 3.97 (1.22)*	− 9.18 (1.30)*	− 5.22 (− 8.63, − 1.80)	**0.003**	− 1.91 (− 5.55, 1.73)	0.308

Estimated means (standard error) of the changes in variables from baseline to month six and between-group differences were derived from the linear mixed-effects model analysis. Model 1 treated group, time, and the group-by-time interaction as the fixed effect factors, and individuals as the random effect factor, while age and sex were controlled as covariates. Model 2 was further adjusted for compliance (compliance score ≥ 3 or compliance score < 3) during the 6-month intervention. *P* values for the effects of the interaction between group and time were examined by linear mixed models. *p* values were marked in bold to indicate a statistically significance (*p<0.05)*

HDL-C, high-density lipoprotein cholesterol; LDL-C, low-density lipoprotein cholesterol; MetS, metabolic syndrome; SAA, Smartphone app-based arm (control group); SADA, Smartphone app-based plus dietitian arm

^*^Statistically significant change from baseline to post-intervention at *p* < 0.05

^a^Data were log-transformed before analysis due to the skewed distribution

Among the 202 individuals with MetS, the reversion rate was 58.4% (59/101) in the SADA group and 45.5% (46/101) in the SAA group (RR: 1.30; 95% confidence interval [CI]: 1.03, 2.44, *p* = 0.047) (Fig. 3c). At the end of the 6-month intervention, among the 310 participants with ≥ 2 MetS components at baseline, a larger proportion of participants in the SADA group had ≥ 2 MetS components reversed than those in the SAA group (28.2% vs*.* 18.0%* p* = 0.037) (Fig. 3d). When individually considered, participants in the SADA group also had a significantly higher reversion rate for low HDL-C levels (*p* = 0.011) and central obesity (*p* = 0.010) compared with those in the SAA group (Fig. 3e). The reversion rates of elevated levels of fasting glucose, triglycerides, and blood pressure were slightly higher in the SADA group than in the SAA group, although the differences were not statistically significant (Fig. 3e). Similar results were also indicated in the sensitivity analysis using per-protocol data (Table S2 and Fig. S2).

### Compliance and Weight Loss

As expected, participants in the SADA group had a much higher engagement with the weight-loss program over the six months than those in the SAA group, as indicated by the five core behaviors, as well as the overall compliance score (all *p* < 0.001) (Table 3, Fig. S3). Participants in the SADA group had more frequent diet monitoring and smart band wearing (*p* < 0.001) (Table 3), which might have enhanced their awareness to restrict food intake and be physically active compared with those in the SAA group, although no significant between-group difference was observed in the changes of energy intake or physical activity (Table S3). Higher compliance for all these core behaviors was positively correlated with larger reductions in body weight, percentage of body fat, change in the visceral area, and waist circumference (*r*_*s*_ =  − 0.23 to − 0.53,* p* < 0.001), accompanied by a larger increase in the percentage of lean mass (*p* < 0.001) (Fig. S4). Among the five core behaviors, the changes in body weight had strongest correlations with the completion of the online learning courses (*r* =  − 0.51, *p* < 0.001), and regular self-weighing (*r* =  − 0.53, *p* < 0.001) (Fig. S4).

**Table 3:** Engagement with the program for participants in SAA and SADA over the 6-month intervention

	All (N = 395)	SAA (N = 198)	SADA (N = 197)	*p*-value
Online courses learning				
Complete sessions	5.0 (0, 39.5)	2.0 (0, 18.5)	11.0 (1.0, 52.0)	** < 0.001**
Compliance, %^a^	5.1 (0, 40.3)	2.0 (0, 18.9)	11.2 (1.0, 53.1)	** < 0.001**
Smart band using				
Compliant days	14.0 (0, 51.5)	5.0 (0, 19.0)	35.0 (7.0, 73.0)	** < 0.001**
Compliance, %^a^	7.8 (0, 28.6)	2.8 (0, 10.6)	19.4 (3.9, 40.6)	** < 0.001**
Weight monitoring				
Compliant weeks	5.0 (0, 16.0)	1.0 (0, 9.0)	9.0 (2.0, 21.0)	** < 0.001**
Compliance, %^b^	20.8 (0, 66.7)	4.2 (0, 37.5)	37.5 (8.3, 87.5)	** < 0.001**
Blood pressure monitoring				
Compliant weeks	0 (0, 3.0)	0 (0, 1.0)	1.0 (0, 5.0)	** < 0.001**
Compliance, %^b^	0 (0, 12.5)	0 (0, 4.2)	4.2 (0, 20.8)	** < 0.001**
Dietary intake recording				
Compliant days	14.0 (4.0, 47.5)	7.0 (3.0, 21.8)	30.0 (11.0, 65.0)	** < 0.001**
Compliance, %^a^	7.8 (2.2, 26.4)	3.9 (1.7, 12.1)	16.7 (6.1, 36.1)	** < 0.001**
Overall compliance score^c^	3 (0, 4)	1 (0, 3)	4 (2, 5)	** < 0.001**

Values were presented as medians (interquartile range) for variables as all compliance variables were identified to be skew distribution. The *p* values were calculated using the Wilcoxon test. Compliance with the intervention was defined as to what extent that participants carried the required five core tasks. *p* values were marked in bold to indicate a statistically significance (*p*<0.05)

SAA, Smartphone app-based arm (control group); SADA, Smartphone app-based plus dietitian arm

^a^Compliance was calculated as the number of self-monitoring days or completed courses as required/168 days or 98 sessions × 100%

^b^Compliance was calculated as the number of weeks fulfilled recommended monitoring frequency/24 weeks × 100%

^c^Participants were assigned one point if their compliance was in the upper half for each behavior; otherwise, zero points. The overall compliance score was calculated by summing the individual score for these five core behaviors, ranging from zero to five, with a higher score indicating better compliance with the intervention program. Because of the generally low frequency of blood pressure monitoring, in the calculation of the overall compliance score, one point was assigned when participants measured their blood pressure once per week instead of three times per week as recommended

Notably, the between-group differences in changes in all outcomes were markedly attenuated by further adjusting for the overall compliance score (Table 2, model 2). When participants had high compliance scores (≥ 3), the significant between-group differences were diminished for most measurements ( Table S4 and Fig. S5), meanwhile the proportion of participants who lost ≥ 10% of their initial weight was similar in the SADA and SAA groups (20.0% vs. 22.1%, *p* = 0.55) (Fig. 2b). Moreover, the trajectory of weight loss over the trial period exhibited a similar trend in these two groups (Fig. S5).

Mediation analysis suggested that the compliance score mediated more than 30% of the between-group difference in weight reduction (*p* < 0.001) (Table 4). After mutual adjustment for the five compliant behaviors, self-weighing was the primary mediator for weight loss, with a mediation proportion of 17.3% –19.2% (*p* < 0.05) (Table 4). The mediation analysis between the intervention and reduced BMI, treating compliance as the potential mediator, showed similar results (Table S5).

**Table 4:** Mediating effect of the intervention compliance on the between-group difference in weight change

	Direct effect (95% CI)	Total effect (95% CI)	Percentage mediation (95% CI)	*p*-value
Weight change, kg				
Compliance score	0.75 (0.27, 1.23)	1.12 (0.65, 1.63)	33.11 (19.20, 57.48)	** < 0.001**
Online courses learning	0.73 (0.30, 1.18)	0.74 (0.31, 1.21)	1.78 (− 6.19, 10.84)	0.610
Weight monitoring	0.73 (0.26, 1.16)	0.88 (0.40, 1.32)	17.28 (3.08, 41.90)	**0.018**
Blood pressure monitoring	0.73 (0.29, 1.15)	0.73 (0.29, 1.15)	− 0.09 (− 0.04, 3.68)	0.920
Smart band using	0.73 (0.29, 1.18)	0.74 (0.30, 1.18)	1.26 (− 4.00,8.58)	0.568
Dietary intake recording	0.73 (0.29, 1.70)	0.73 (0.31, 1.18)	0.00 (− 4.48, 4.28)	1.000
Weight changes, %				
Compliance score	0.90 (0.21, 1.54)	1.36 (0.65, 2.01)	33.94 (17.89, 70.63)	** < 0.001**
Online courses learning	0.85 (0.17, 1.52)	0.87 (0.18, 1.54)	2.20 (− 12.61, 15.75)	0.618
Weight monitoring	0.88 (0.22, 1.50)	1.09 (0.42, 1.73)	19.24 (3.05, 53.10)	**0.026**
Blood pressure monitoring	0.88 (0.21, 1.51)	0.88 (0.21, 1.51)	− 0.25 (− 5.48, 4.54)	0.814
Smart band using	0.87 (0.23, 1.52)	0.86 (0.23, 1.50)	− 0.40 (− 8.19, 6.79)	0.838
Dietary intake recording	0.87 (0.19, 1.51)	0.86 (0.19, 1.50)	− 0.65 (− 6.81, 3.09)	0.562

The mediation analysis was conducted based on all available data which was tracked weekly during the 6-month intervention period among all randomized participants with blue-tooth devices. The linear mixed models were adopted for the mediation analysis including intervention group, time (by week), and their interaction as fixed effect factors and individual as the random effect factor, adjusting for age, gender, current smoking and drinking, physical activity, educational level, and energy intake at baseline. Intervention compliance refers to the overall compliance score or the compliance of each behavior calculated for each week. *p* values were marked in bold to indicate a statistically significance (*p*<0.05)

### Determinants of Compliance

In multivariate analysis, the factors that impacted both individual and collective behavioral compliance were analyzed, including baseline characteristics (age, sex, educational level, smoking, drinking, body weight, BMI, waist circumference, and the number of MetS components), weight loss in the initial two weeks, self-perceived motivation, barriers, simplicity, and satisfaction with the overall intervention program. As shown in Table 5, having ≥ 10 years of educational experience was significantly associated with better compliance of three behaviors (*p* < 0.05) and the overall compliance score (*p* = 0.002), but were not associated with blood pressure monitoring or wearing the smart band. Greater weight loss in the initial two weeks was associated with a higher frequency of online learning and self-weighing, as well as a higher total compliance score (all *p* < 0.05). Self-perceived satisfaction was significantly associated with behavioral compliance, such as completing online learning courses, recording dietary intake, and the overall compliance score (*p* < 0.01), while individuals who felt that it was easy to follow the instructions tended to monitor their weight and blood pressure and wear the smart band more frequently (*p* < 0.05).

**Table 5:** Factors associated with intervention adherence

	Compliance score		Online courses learning		Weight monitoring		Blood pressure monitoring		Smart band using		Dietary intake recording	
	OR	*p*-value	OR	*p*-value	OR	*p*-value	OR	*p*-value	OR	*p*-value	OR	*p*-value
Age	–	–	–	–	–	–	–	–	–	–	–	–
Gender	–	–	–	–	–	**–**	–	**–**	–	**–**	–	–
Education (≥ 10 years)	2.39	**0.013**	3.49	**0.005**	3.25	**0.006**	–	**–**	1.87	0.078	2.66	**0.008**
Current smoker	–	**–**	–	–	–	–	–	**–**	–	**–**	–	**–**
Alcohol drinker	–	**–**	–	–	–	–	–	**–**	–	–	–	**–**
Body weight at baseline	–	**–**	–	–	–	–	–	**–**	–	**–**	–	**–**
Body-mass index at baseline	–	**–**	–	–	–	–	–	**–**	–	**–**	–	**–**
Waist circumference at baseline	–	**–**	–	**–**	–	**–**	–	–	–	**–**	–	**–**
The number of Mets risk factors at baseline	–	–	–	–	–	–	–	–	–	–	–	–
Percentage of body weight loss in first two weeks	1.14	**0.022**	1.25	**0.002**	1.22	**0.006**	–	**–**	–	**–**	–	**–**
Motivation	–	–	–	–	–	–	–	–	–	–	–	–
Barrier	–	–	–	–	–	–	–	–	–	–	–	–
Simplicity	–	–	–	–	1.02	**0.006**	1.01	**0.020**	1.02	**0.001**	–	–
Satisfaction	1.02	** < 0.001**	1.02	**0.002**	–	–	–	–	–	–	1.02	** < 0.001**

The table includes variables that remained significant or had a considerable effect (0.05 < *p*-value < 0.1) in either the multivariate logistic models of individual behavioral compliance or the multilevel logistic model for the overall compliance score after the backward stepwise selection procedures. *p* values were marked in bold to indicate a statistically significance (*p*<0.05)

“–” denotes factors tested but did not remain in the final model after the backward stepwise selection procedures**.** Abbreviations: OR, odds ratio

## Discussion

In our 6-month RCT including overweight/obese or centrally obese Chinese individuals, although the lifestyle intervention led to significant weight loss and better metabolic profiles in the SADA group than in the SAA group, the between-group difference was considerably attenuated when participants had a compliance score ≥ 3 and even disappeared for those who lost ≥ 10% of their initial weight. Over 30% of the between-group difference in weight reduction was mediated by their behavioral compliance, and self-weighing was the primary mediator. Potential determinants of adherence also included higher levels of education and initial weight loss, self-perceived simplicity, and satisfaction with the intervention program.

By adopting the app-based lifestyle intervention for six months, the participants in the SAA group lost approximately 1.82 kg of their initial weight, which is within the previously reported ranges of 0.03–3.9 kg (Beleigoli et al. 2020; Eisenhauer et al. 2021; Laing et al. 2014) and 0.70–2.8 kg weight reduction in a recent meta-analysis of app-based interventions, which were conducted mostly in Western populations (Antoun et al. 2022). On the other hand, when integrating human coaches into online programs, the SADA group showed a weight loss of approximately 3.59 kg, comparable to the findings from most Western population-based studies that reported weight reductions of 1.57–5.11 kg (Baer et al. 2020; Beleigoli et al. 2020; Chang et al. 2022; Lim et al. 2021). Indeed, our participants had lower levels of adiposity (BMI < 28 kg/m^2^) than those in Western studies often with higher BMIs (≥ 30 kg/m^2^), which tended to observe greater weight loss (Ryan et al. 2017). In the case of MetS or its components, participants in SADA tended to have more favorable changes, and within-group changes in circulating triglyceride and HDL-C levels reached statistical significance. A study in the Asian population adopting dietitian coaching reported significant glycemic and lipid improvements, while only systolic blood pressure was found to decrease in another trial among Germans (Brame et al. 2022; Lim et al. 2021). These discrepancies in intervention effects among studies may be attributed to the heterogeneity in the studies with differences in participant characteristics, study design (e.g., app design, trial durations, and intervention program components), and habitual background lifestyles. It also remains to be elucidated whether the lack of vast improvement in MetS and its components could be attributed to the fact that only 45.38% of participants in SADA had ≥ 5% weight loss, regardless this proportion is slightly greater than the finding in another recent web-based intervention (Kohl et al. 2023).

As a crucial influencer for successful weight loss, behavioral compliance could be promoted by professional support (Kupila et al. 2023; Mohr et al. 2011), in agreement with the fact that the SADA group showed greater weight reduction via improved compliance, as well as the findings from a study conducted by Farage et al. who suggested that the compliance with app use and self-weighing mediated over 50% of the relationship between treatment allocation and weight loss in the first four months among participants from the U.S. military (Farage et al. 2021). Indeed, our participants in SADA received intensive coaching from an online dietitian regarding the knowledge and skills to monitor and regulate their weight by behavioral modifications based on self-monitoring data, which may enhance compliance (Teixeira et al. 2015). Furthermore, we observed that frequent weighing appeared to be the most important mediator linked with weight loss outcomes compared with other self-monitoring strategies and has been recommended for weight loss and weight maintenance (Wing et al. 2006). Self-weighing as a behavior modification could contribute to the awareness of weight fluctuations via instant feedback. By applying the same platform of app-connected weight scales in our recent full-feeding trial, a 25% calorie restriction for 6 months resulted in a mean weight loss of 7% (5.5 kg), which could be partly explained by monitoring weight changes and consequently modifying dietary intake (Luo et al. 2022). We noticed that when analyzing participants with high compliance scores (≥ 3), the two groups had a similar proportion of participants who achieved clinically meaningful weight loss, especially in those who had weight loss ≥ 10% following the 6-month intervention. On the other hand, in a recent meta-analysis, when combined with a fully automated intervention, individuals who received less intensive additional coaching exhibited greater weight loss than those who received higher-intensity coaching (Berry et al. 2021). Thus, identifying potential highly compliant individuals and providing relatively low-intensity professional support might be more relevant and cost-effective for weight loss and maintenance among well-disciplined participants.

When multiple determinants of compliance were considered, we found that participants with higher educational attainment were more likely to engage than their counterparts with lower educational attainment. Obviously, persons with high education levels could better understand the recommendations of the lifestyle intervention program and more easily communicate with and trust dietitians (Chen et al. 2022). They were more sophisticated in handling the smartphone app-based intervention, which was particularly important. Moreover, our multivariate analysis also revealed that adherent participants felt that it was easier to follow the trial instructions and were more satisfied with the intervention program. Thus, they were more comfortable accomplishing all assigned tasks and obtained higher self-efficacy. In addition, initial weight loss at an early stage was suggested to influence long-term adherence to the lifestyle intervention program (Greenberg et al. 2009; Miller et al. 2015). We also found that greater weight loss in the initial two weeks was associated with higher compliance scores, more frequent online learning, and self-weighing. Identifying potential determinants can facilitate not only the identification of highly motivated and disciplined individuals but also the development of more targeted approaches, such as the use of app-based interventions only or the combination of low-intensity interventions with dietitian aids according to the major determinants.

To our knowledge, this study is the first study to systematically assess the dynamic of behavioral phenotypes and quantify the role(s) of behavioral compliance (both individually and collectively) in a 6-month smartphone app-based lifestyle intervention trial. Unlike most existing lifestyle intervention studies that relied on self-reported data of weight tracking or compliance, our study adopted app-connected multiple devices such as Bluetooth scales and smart bands to promote self-monitoring and adherence over six months. Admittedly, there were some limitations. First, our intervention trial only lasted six months due to limited resources, and it would be of interest to follow the participants for longer than one year to evaluate the long-term effectiveness of weight maintenance following the lifestyle intervention. Second, the scalability of this multi-component intervention could not be evaluated without a cost-effectiveness analysis, although similar smartphone approaches have been proven to be cost-effective when compared to conventional face-to-face interventions (Little et al. 2016). Furthermore, given the nature of the lifestyle intervention, blinding participants and personnel was difficult. The expectations of participants and dietitians could influence the implementation of the intervention, despite assessors being blinded to the allocation and hypothesis in this study.

## Conclusion

In this study, we found stronger effects of the smartphone app plus online dietitian support than the smart app-based lifestyle intervention alone regarding weight loss and improved metabolic outcomes. The between-group differences were diminished by high compliance with the core behaviors, especially for those with regular self-weighing, and some demographic and intervention response features of the participants also influenced their behavioral adherence to some extent. The results of our study are informative for the future development of more precise and cost-effective strategies in smartphone app-based long-term weight management. However, more trials are needed to confirm our findings.

## Supplementary Information

Below is the link to the electronic supplementary material.

Supplementary file 1 (DOCX 10541 kb)

## Data Availability

The data that support the findings of this study are not openly available due to reasons of sensitivity and are available from the corresponding authors upon reasonable request.

## Abbreviations

AHLQ: Adherence to a Healthy Lifestyle Questionnaire
BMI: Body mass index
CI: Confidence interval
DPP: Diabetes Prevention Program
EDTA: Ethylenediaminetetra-acetic acid
GI: Glycemic index
HDL-C: High-density lipoprotein cholesterol
IQR: Interquartile range
ITT: Intent-to-treat
LDL-C: Low-density lipoprotein cholesterol
NCEP ATPIII: National Cholesterol Education Program Adult Treatment Panel III
PASS: Power Analysis & Sample Size software
*r*_*s*_: Spearman correlation coefficients
SAA: Smartphone app-based arm
SADA: Smartphone app plus dietitian arm
MetS: Metabolic syndrome
RCT: Randomized control trial
RR: Relative rate
SAS: Statistical analysis system software
SD: Standard deviation

## Declarations

### Registration

This trial was registered at ClinicalTrials.gov (NCT04714723) on January 14, 2021.

## References

[CR1] 50th China Statistical Report on Internet Development (2022). https://www.cnnic.com.cn/IDR/ReportDownloads/

[CR2] Abarca-Gómez L, Abdeen ZA, Hamid ZA et al (2017) Worldwide trends in body-mass index, underweight, overweight, and obesity from 1975 to 2016: a pooled analysis of 2416 population-based measurement studies in 128.9 million children, adolescents, and adults. Lancet 390(10113):2627–2642. 10.1016/S0140-6736(17)32129-3 29029897 10.1016/S0140-6736(17)32129-3 PMC5735219

[CR3] Alberti KG, Eckel RH, Grundy SM et al (2009) Harmonizing the metabolic syndrome: a joint interim statement of the International Diabetes Federation Task Force on Epidemiology and Prevention; National Heart, Lung, and Blood Institute; American Heart Association; World Heart Federation; International Atherosclerosis Society; and International Association for the Study of Obesity. Circulation 120(16):1640–1645. 10.1161/circulationaha.109.192644 19805654 10.1161/CIRCULATIONAHA.109.192644

[CR4] Ang SM, Chen J, Liew JH et al (2021) Efficacy of interventions that incorporate mobile apps in facilitating weight loss and health behavior change in the asian population: systematic review and meta-analysis. J Med Internet Res 23(11):e28185. 10.2196/28185 34783674 10.2196/28185 PMC8663646

[CR5] Antoun J, Itani H, Alarab N et al (2022) The effectiveness of combining nonmobile interventions with the use of smartphone apps with various features for weight loss: systematic review and meta-analysis. JMIR Mhealth Uhealth 10(4):e35479. 10.2196/35479 35394443 10.2196/35479 PMC9034427

[CR6] Association AD (2020) 7. Diabetes technology: standards of medical care in diabetes—2021. Diabetes Care 44(Supplement_1):S85–S99. 10.2337/dc21-S007 10.2337/dc21-S007 33298418

[CR7] Baer HJ, Rozenblum R, De La Cruz BA et al (2020) Effect of an online weight management program integrated with population health management on weight change: a randomized clinical trial. JAMA 324(17):1737–1746. 10.1001/jama.2020.18977 33141209 10.1001/jama.2020.18977 PMC7610192

[CR8] Beleigoli A, Andrade AQ, Diniz MF et al (2020) Personalized web-based weight loss behavior change program with and without dietitian online coaching for adults with overweight and obesity: randomized controlled trial. J Med Internet Res 22(11):e17494. 10.2196/17494 33151151 10.2196/17494 PMC7677024

[CR9] Berry MP, Sala M, Abber SR et al (2021) Incorporating automated digital interventions into coach-delivered weight loss treatment: a meta-analysis. Health Psychol 40(8):534–545. 10.1037/hea0001106 34618500 10.1037/hea0001106

[CR10] Bhaskaran K, Douglas I, Forbes H et al (2014) Body-mass index and risk of 22 specific cancers: a population-based cohort study of 5·24 million UK adults. Lancet 384(9945):755–765. 10.1016/s0140-6736(14)60892-8 25129328 10.1016/S0140-6736(14)60892-8 PMC4151483

[CR11] Brame J, Centner C, Berg N et al (2022) Effects of a 12-week web-based weight loss program for adults with overweight and obesity on COVID age and lifestyle-related cardiometabolic risk factors: a randomized controlled trial. Front Public Health 10:868255. 10.3389/fpubh.2022.868255 35669738 10.3389/fpubh.2022.868255 PMC9163343

[CR12] Bray GA, Frühbeck G, Ryan DH et al (2016) Management of obesity. Lancet 387(10031):1947–1956. 10.1016/s0140-6736(16)00271-3 26868660 10.1016/S0140-6736(16)00271-3

[CR13] Bray GA, Kim KK, Wilding JPH et al (2017) Obesity: a chronic relapsing progressive disease process. A position statement of the World Obesity Federation. Obes Rev 18(7):715–723. 10.1111/obr.12551 28489290 10.1111/obr.12551

[CR14] Chang AR, Gummo L, Yule C et al (2022) Effects of a dietitian-led, telehealth lifestyle intervention on blood pressure: results of a randomized. Controlled Trial J Am Heart Assoc 11(19):e027213. 10.1161/jaha.122.027213 36172955 10.1161/JAHA.122.027213 PMC9673709

[CR15] Chen JN, Dennis JA, St John JA et al (2022) Self-reported patient compliance with physician advised lifestyle behavior changes among adults with musculoskeletal conditions. Front Public Health 10:821150. 10.3389/fpubh.2022.821150 35284362 10.3389/fpubh.2022.821150 PMC8907563

[CR16] Chew HSJ, Koh WL, Ng J et al (2022) Sustainability of weight loss through smartphone apps: systematic review and meta-analysis on anthropometric, metabolic, and dietary outcomes. J Med Internet Res 24(9):e40141. 10.2196/40141 36129739 10.2196/40141 PMC9536524

[CR17] Chopra S, Malhotra A, Ranjan P et al (2021) Predictors of successful weight loss outcomes amongst individuals with obesity undergoing lifestyle interventions: a systematic review. Obes Rev 22(3):e13148. 10.1111/obr.13148 33200547 10.1111/obr.13148

[CR18] Craig CL, Marshall AL, Sjöström M et al (2003) International physical activity questionnaire: 12-country reliability and validity. Med Sci Sports Exerc 35(8):1381–1395. 10.1249/01.Mss.0000078924.61453.Fb 12900694 10.1249/01.MSS.0000078924.61453.FB

[CR19] Diabetes Prevention Program (DPP) Research Group (2002) The Diabetes Prevention Program (DPP): description of lifestyle intervention. Diabetes Care 25(12):2165–2171. 10.2337/diacare.25.12.2165 10.2337/diacare.25.12.2165 PMC1282458 12453955

[CR20] Eisenhauer CM, Brito F, Kupzyk K et al (2021) Mobile health assisted self-monitoring is acceptable for supporting weight loss in rural men: a pragmatic randomized controlled feasibility trial. BMC Public Health 21(1):1568. 10.1186/s12889-021-11618-7 34407782 10.1186/s12889-021-11618-7 PMC8375071

[CR21] Fan JG, Kim SU, Wong VW (2017) New trends on obesity and NAFLD in Asia. J Hepatol 67(4):862–873. 10.1016/j.jhep.2017.06.003 28642059 10.1016/j.jhep.2017.06.003

[CR22] Farage G, Simmons C, Kocak M et al (2021) Assessing the contribution of self-monitoring through a commercial weight loss app: mediation and predictive modeling study. JMIR Mhealth Uhealth 9(7):e18741. 10.2196/18741 34259635 10.2196/18741 PMC8319781

[CR23] Ghaben AL, Scherer PE (2019) Adipogenesis and metabolic health. Nat Rev Mol Cell Biol 20(4):242–258. 10.1038/s41580-018-0093-z 30610207 10.1038/s41580-018-0093-z

[CR24] Greenberg I, Stampfer MJ, Schwarzfuchs D et al (2009) Adherence and success in long-term weight loss diets: the dietary intervention randomized controlled trial (DIRECT). J Am Coll Nutr 28(2):159–168. 10.1080/07315724.2009.10719767 19828901 10.1080/07315724.2009.10719767

[CR25] Group TDPPR (2002) The Diabetes Prevention Program (DPP): description of lifestyle intervention. Diabetes Care 25(12):2165–2171. 10.2337/diacare.25.12.2165 12453955 10.2337/diacare.25.12.2165 PMC1282458

[CR26] Haththotuwa RN, Wijeyaratne CN, Senarath U (2020) Worldwide epidemic of obesity. In: Obesity and obstetrics. Elsevier, London, pp 3–8

[CR27] Jensen MD, Ryan DH, Apovian CM et al (2014) 2013 AHA/ACC/TOS guideline for the management of overweight and obesity in adults: a report of the American College of Cardiology/American Heart Association Task Force on Practice Guidelines and The Obesity Society. Circulation 63(25):2985–3023 10.1016/j.jacc.2013.11.004 24239920

[CR28] Kohl J, Brame J, Centner C et al (2023) Effects of a web-based lifestyle intervention on weight loss and cardiometabolic risk factors in adults with overweight and obesity: randomized controlled clinical trial. J Med Internet Res 25:e43426. 10.2196/43426 37368484 10.2196/43426 PMC10337343

[CR29] Kupila SKE, Joki A, Suojanen L-U et al (2023) The effectiveness of ehealth interventions for weight loss and weight loss maintenance in adults with overweight or obesity: a systematic review of systematic reviews. Curr Obes Rep. 10.1007/s13679-023-00515-2 37354334 10.1007/s13679-023-00515-2 PMC10482795

[CR30] Laing BY, Mangione CM, Tseng C-H et al (2014) Effectiveness of a smartphone application for weight loss compared with usual care in overweight primary care patients. Ann Intern Med 161(Supplement_10):S5–S12. 10.7326/M13-3005 25402403 10.7326/M13-3005 PMC4422872

[CR31] Lim SL, Ong KW, Johal J et al (2021) Effect of a smartphone app on weight change and metabolic outcomes in asian adults with type 2 diabetes: a randomized clinical trial. JAMA Netw Open 4(6):e2112417. 10.1001/jamanetworkopen.2021.12417 34081137 10.1001/jamanetworkopen.2021.12417 PMC8176331

[CR32] Little P, Stuart B, Hobbs FDR et al (2016) An internet-based intervention with brief nurse support to manage obesity in primary care (POWeR+): a pragmatic, parallel-group, randomised controlled trial. Lancet Diabetes Endocrinol 4(10):821–828. 10.1016/S2213-8587(16)30099-7 27474214 10.1016/S2213-8587(16)30099-7

[CR33] Luo Y, Wang J, Sun L et al (2022) Isocaloric-restricted Mediterranean Diet and Chinese diets high or low in plants in adults with prediabetes. J Clin Endocrinol Metab 107(8):2216–2227. 10.1210/clinem/dgac303 35579171 10.1210/clinem/dgac303 PMC9282247

[CR34] Malekzadeh S, Chong MC, Tang LY et al (2022) Effectiveness of an educational program through mobile health application on knowledge and adherence to a healthy lifestyle in cardiac syndrome X patients. Iran Red Crescent Med J. 10.32592/ircmj.2022.24.6.1803

[CR35] Miller CK, Nagaraja HN, Weinhold KR (2015) Early weight-loss success identifies nonresponders after a lifestyle intervention in a worksite diabetes prevention trial. J Acad Nutr Diet 115(9):1464–1471. 10.1016/j.jand.2015.04.022 26095435 10.1016/j.jand.2015.04.022 PMC4554978

[CR36] Mohr DC, Cuijpers P, Lehman K (2011) Supportive accountability: a model for providing human support to enhance adherence to eHealth interventions. J Med Internet Res 13(1):e30. 10.2196/jmir.1602 21393123 10.2196/jmir.1602 PMC3221353

[CR37] Muramoto A, Matsushita M, Kato A et al (2014) Three percent weight reduction is the minimum requirement to improve health hazards in obese and overweight people in Japan. Obes Res Clin Pract 8(5):e466–e475. 10.1016/j.orcp.2013.10.003 25263836 10.1016/j.orcp.2013.10.003

[CR38] Pagoto S (2011) The current state of lifestyle intervention implementation research: where do we go next? Transl Behav Med 1(3):401–405. 10.1007/s13142-011-0071-x 24073065 10.1007/s13142-011-0071-x PMC3717623

[CR39] Pan X-F, Wang L, Pan A (2021) Epidemiology and determinants of obesity in China. Lancet Diabetes Endocrinol 9(6):373–392. 10.1016/S2213-8587(21)00045-0 34022156 10.1016/S2213-8587(21)00045-0

[CR40] Powell-Wiley TM, Poirier P, Burke LE et al (2021) Obesity and cardiovascular disease: a scientific statement from the American Heart Association. Circulation 143(21):e984–e1010. 10.1161/CIR.0000000000000973 33882682 10.1161/CIR.0000000000000973 PMC8493650

[CR41] Ryan DH, Yockey SR (2017) Weight loss and improvement in comorbidity: differences at 5%, 10%, 15%, and over. Curr Obes Rep 6(2):187–194. 10.1007/s13679-017-0262-y 28455679 10.1007/s13679-017-0262-y PMC5497590

[CR42] Serrano KJ, Yu M, Coa KI et al (2016) Mining health app data to find more and less successful weight loss subgroups. J Med Internet Res 18(6):e154. 10.2196/jmir.5473 27301853 10.2196/jmir.5473 PMC4925935

[CR43] Teixeira PJ, Carraça EV, Marques MM et al (2015) Successful behavior change in obesity interventions in adults: a systematic review of self-regulation mediators. BMC Med 13(1):84. 10.1186/s12916-015-0323-6 25907778 10.1186/s12916-015-0323-6 PMC4408562

[CR44] The Mobile Economy Asia Pacific (2020) G association. https://www.gsma.com/mobileeconomy/wp-content/uploads/2020/06/GSMA_MobileEconomy_2020_AsiaPacific.pdf

[CR45] Thomas JG, Leahey TM, Wing RR (2015) An automated internet behavioral weight-loss program by physician referral: a randomized controlled trial. Diabetes Care 38(1):9–15. 10.2337/dc14-1474 25404659 10.2337/dc14-1474 PMC4274778

[CR46] Tingley D, Yamamoto T, Hirose K et al (2014) Mediation: R package for causal mediation analysis. J Stat Softw 59(5):1–38. 10.18637/jss.v059.i0526917999

[CR47] van Buuren S, Groothuis-Oudshoorn K (2011) Mice: multivariate imputation by chained equations in R. J Stat Softw 45(3):1–67. 10.18637/jss.v045.i03

[CR48] Venables WN, Ripley BD (2002) Modern applied statistics with S, vol 4th edn. Springer. https://www.stats.ox.ac.uk/pub/MASS4/

[CR49] Wadden TA, Tronieri JS, Butryn ML (2020) Lifestyle modification approaches for the treatment of obesity in adults. Am Psychol 75(2):235–251. 10.1037/amp0000517 32052997 10.1037/amp0000517 PMC7027681

[CR50] Wang L, Zhou B, Zhao Z et al (2021) Body-mass index and obesity in urban and rural China: findings from consecutive nationally representative surveys during 2004–18. Lancet 398(10294):53–63. 10.1016/s0140-6736(21)00798-4 34217401 10.1016/S0140-6736(21)00798-4 PMC7617101

[CR51] Wing RR, Tate DF, Gorin AA et al (2006) A self-regulation program for maintenance of weight loss. New Engl J Med 355(15):1563–1571. 10.1056/NEJMoa061883 17035649 10.1056/NEJMoa061883

[CR52] Wing RR, Lang W, Wadden TA et al (2011) Benefits of modest weight loss in improving cardiovascular risk factors in overweight and obese individuals with type 2 diabetes. Diabetes Care 34(7):1481–1486. 10.2337/dc10-2415 21593294 10.2337/dc10-2415 PMC3120182

[CR53] Wu H, Pan A, Yu Z et al (2010) Lifestyle counseling and supplementation with flaxseed or walnuts influence the management of metabolic syndrome. J Nutr 140(11):1937–1942. 10.3945/jn.110.126300 20826632 10.3945/jn.110.126300 PMC3361016

[CR54] Ye X, Yu Z, Li H et al (2007) Distributions of C-reactive protein and its association with metabolic syndrome in middle-aged and older Chinese people. J Am Coll Cardiol 49(17):1798–1805. 10.1016/j.jacc.2007.01.065 17466231 10.1016/j.jacc.2007.01.065

[CR55] Zhu D (2021) Guideline for the prevention and treatment of type 2 diabetes mellitus in China (2020 edition). Chin J Pract Intern Med 41:668–695

