# Supplementary material for: Impacts of Behavioral Compliance on Weight-Loss and Metabolic Profile in a Smartphone App-Based Lifestyle Intervention or Plus Dietitian Supporting: A Randomized and Controlled Trial Among Chinese
Source: Phenomics. 2025 Jan 30;5(2):154–68. doi: 10.1007/s43657-024-00162-0 (PMC12209060; doi:10.1007/s43657-024-00162-0)
Supplement: Supplementary file 1 — (DOCX 10541 kb) [file 43657_2024_162_MOESM1_ESM.docx]

**Impacts of Behavioral Compliance on Weight-Loss and Metabolic Profile in a Smartphone App-based Lifestyle Intervention or Plus Dietitian Supporting: A Randomized and Controlled Trial among Chinese**

**Authors:** Xue Li^1*^, Ling Lu^1*^, Liang Sun^2^, Yunxia Xie^3^, Kang Huang^1^, Changzheng Yuan^4,5^, Liying Chen^6, #^, Xu Lin^1, 7, #^

**^*^** Co-first authors, contributed equally.

^#^ Contributed equally as senior authors.

**Emails and ORCIDs**

Xue Li: xueli@ucas.ac.cn; 0000-0002-6576-9182; Ling Lu: luling@ucas.ac.cn; 0000-0002-1670-5027; Liang Sun: sun_liang@fudan.edu.cn; 0000-0002-5554-4801; Yunxia Xie: yayakii@126.com; Kang Huang: huangkang22@mails.ucas.ac.cn; 0009-0005-4459-3543; Changzheng Yuan: chy478@zju.edu.cn; 0000-0002-2389-8752; Liying Chen: 3197020@zju.edu.cn; 0000-0002-8895-3775; Xu Lin: xlin@sibs.ac.cn; 0000-0001-8876-9823

**Affiliations:**

^1^ Key Laboratory of Systems Health Science of Zhejiang Province, School of Life Science, Hangzhou Institute for Advanced Study, University of Chinese Academy of Sciences, Hangzhou 310024, China

^2^ Ministry of Education Key Laboratory of Public Health Safety, School of Public Health, Institute of Nutrition, Fudan University, Shanghai 200032, China

^3^ Nursing department, Sir Run Run Shaw Hospital, Zhejiang University School of Medicine, Hangzhou 310016, China

^4^ School of Public Health, the Second Affiliated Hospital, Zhejiang University School of Medicine, Hangzhou 310058, China.

^5^ Department of Nutrition, Harvard T.H. Chan School of Public Health, Boston 02115, USA.

^6^ Department of General Practice, Sir Run Run Shaw Hospital, School of Medicine, Zhejiang University, Hangzhou 310016, China

^7^ Shanghai Institute of Nutrition and Health, University of Chinese Academy of Sciences, Chinese Academy of Sciences, Shanghai 200031, China.

**Corresponding Authors**

Xu Lin, Shanghai Institute of Nutrition and Health, Chinese Academy of Sciences, 320 Yue-yang Rd., Shanghai 200031, China; Hangzhou Institute for Advanced Study, University of Chinese Academy of Sciences, Chinese Academy of Sciences, Hangzhou 310024, China. Email: xlin@sibs.ac.cn;

Liying Chen, Department of General Practice, Sir Run Run Shaw Hospital, School of Medicine, Zhejiang University, Hangzhou 310016, China. Email: 3197020@zju.edu.cn

**SUPPLEMENTAL METHODS**

**Inclusion and exclusion criteria**

Participants were included if they were 20-65 years of age, with overweight/obesity (BMI ≥24 kg/m^2^, defined by the Chinese criteria(Pan et al. 2021)), or central obesity (defined as a waistline ≥ 90 cm for males or ≥ 80 cm for females). The exclusion criteria included: (1) fasting glucose ≥7.8 mmol/L even when using hypoglycemic medications; (2) clinically diagnosed stage III hypertension(Jones et al. 2020) and the systolic blood pressure (SBP) ≥140mmHg when using antihypertensive drugs; (3) fasting plasma triglycerides≥5.7 mmol/L or fasting plasma LDL-c≥4.9mmol/L when using lipid lowering medication; (4) pregnancy or lactation; (5) Obvious change of drug usage in three months before enrollment; (6) heavy alcohol use (≥80 g/day for male or ≥40 g/day for female) or other substance abuse; (7) diseases that would affect metabolism condition, i.e. severe kidney, liver, cardiovascular, gastrointestinal, pituitary or thyroid diseases; (8) received surgeries within one year before research (e.g., heart stents implanted surgery and gastrointestinal surgery); (9) cancer or receiving radiotherapy and chemotherapy within five years; (10) suffering from infectious diseases at recruitment; (11) mental disorders impacting ability to participate in the study; (12) participating in other studies in last three months before enrollment.

**Metabolic syndrome definition**

MetS was diagnosed as three or more of the following five factors as defined by the updated National Cholesterol Education Program’s Adult Treatment Panel III (NCEP ATPIII) criteria for Asians(Alberti et al. 2009): 1) central obesity (waist circumference ≥90 cm in men or ≥80 cm in women); 2) triglycerides ≥1.70 mmol/L or on triglycerides treatment; 3) HDL cholesterol <1.03 mmol/L in men or <1.30 mmol/L in women or on HDL-C treatment; 4) fasting glucose ≥5.6 mmol/L or diabetes; 5) systolic blood pressure ≥130 mmHg or diastolic blood pressure ≥85 mmHg or on hypertension treatment.

**Table S1 Baseline characteristics of participants who completed the 6-month intervention versus those who dropped out**

| **Characteristics** | **Completers (n = 347)** | **Dropouts (n = 48)** | ***p*-value** |
| --- | --- | --- | --- |
| Age | 43.7(8.75) | 43.1(10.04) | 0.67 |
| Female, n (%) | 166(47.8) | 22(45.8) | 0.92 |
| Education ≥10 y, no. (%) | 301(86.7) | 41(85.4) | 0.89 |
| Current smoker, no. (%) | 47(13.6) | 3(6.25) | 0.23 |
| Alcohol drinker, no. (%) | 207(59.8) | 30(62.5) | 0.84 |
| Body weight, kg | 75.2(11.3) | 75.5(12.6) | 0.86 |
| BMI, kg/m^2^ | 27.6(2.74) | 27.8(3.18) | 0.71 |
| Overweight/obese, no. (%) | 334 (96.3) | 46 (95.8) | 1.00 |
| Waist circumference, cm | 93.4(7.85) | 93.7(8.77) | 0.79 |
| Body fat mass percentage, % | 33.7(6.25) | 34.4(6.60) | 0.46 |
| Body lean mass percentage, % | 62.6(5.98) | 62.0(6.29) | 0.48 |
| Visceral fat area, cm^2^ | 116.7(21.25) | 118.6(22.28) | 0.55 |
| MetS, no. (%) | 179 (51.6) | 23 (47.9) | 0.75 |
| Elevated fasting glucose | 171 (49.4) | 25 (52.1) | 0.85 |
| Low HDL-C | 161 (46.4) | 17 (35.4) | 0.20 |
| Elevated triglycerides | 130 (37.7) | 15 (31.3) | 0.48 |
| Central obesity | 310 (89.3) | 43 (89.6) | 1.00 |
| Elevated blood pressure | 116 (33.4) | 19 (39.6) | 0.50 |
| ≥2 MetS risk factors, no. (%) | 271 (78.1) | 39 (81.3) | 0.76 |
| Systolic blood pressure, mmHg | 119.4(15.0) | 120.1(13.4) | 0.76 |
| Diastolic blood pressure, mmHg | 79.9(9.83) | 80.0(9.91) | 0.96 |
| Fasting glucose, mmol/L | 5.64(0.56) | 5.69(0.55) | 0.51 |
| Triglycerides mmol/L^a^ | 1.45(1.05, 1.99) | 1.36(1.06, 2.11) | 0.97 |
| Total cholesterol mmol/L | 5.17(1.03) | 4.95(0.95) | 0.17 |
| HDL-c mmol/L | 1.21(0.29) | 1.22(0.29) | 0.76 |
| LDL-c mmol/L | 3.20(0.91) | 2.97(0.84) | 0.097 |

Values were presented as means (standard deviation) for continuous variables (unless indicated otherwise) and as numbers (%) for categorical variables. *P*-values were calculated using the Student’s t-test and the χ2 test for continuous variables and categorical variables, respectively.

^a^ Values were presented as median (interquartile range) due to the skewed distribution.

Abbreviations: HDL-c, high-density lipoprotein cholesterol; LDL-c, low-density lipoprotein cholesterol; MetS, metabolic syndrome.

**Table S2** **Changes of anthropometrics and metabolic risk factors of participants from baseline to month six in the per-protocol analysis**

|  | **Changes over 6-month intervention (SE)** | | **Between-group difference**  **model 1(95% CI)** | ***p*-value** | **Between-group difference**  **model 2 (95% CI)** | ***p*-value** |
| --- | --- | --- | --- | --- | --- | --- |
|  | **SAA (n=172)** | **SADA (n=175)** |  |  |  |  |
| Body weight, kg | -1.72 (0.29)^*^ | -3.67 (0.29)^*^ | -1.95 (-2.75, -1.15) | **<0.001** | -0.84 (-1.63, -0.06) | **0.035** |
| Weight change, % | -2.26(0.38)^*^ | -4.96(0.37)^*^ | -2.66 (-3.71, -1.61) | **<0.001** | -1.26 (-2.30, -0.23) | **0.017** |
| BMI, kg/m^2^ | -0.65 (0.11)^*^ | -1.37 (0.10)^*^ | -0.72 (-1.01, -0.43) | **<0.001** | -0.31 (-0.59, -0.03) | **0.033** |
| Waist circumference, cm | -3.63 (0.37)^*^ | -5.91 (0.37)^*^ | -2.28 (-3.31, -1.26) | **<0.001** | -0.87 (-1.88, 0.15) | 0.093 |
| Systolic blood pressure, mmHg | -2.21 (0.73)^*^ | -2.51 (0.73)^*^ | -0.30 (-2.32, 1.73) | 0.78 | 0.65 (-1.49, 2.80) | 0.55 |
| Diastolic blood pressure, mmHg | -0.91 (0.54) | -2.29 (0.53)^*^ | -1.38 (-2.86, 0.10) | 0.067 | -0.27 (-1.81, 1.28) | 0.73 |
| Fasting glucose, mmol/L | 0.02 (0.03) | -0.04 (0.03) | -0.07 (-0.16, 0.03) | 0.15 | -0.04 (-0.15, 0.06) | 0.44 |
| Triglycerides mmol/L^a^ | -0.01 (0.03) | -0.16 (0.03)^*^ | -0.15 (-0.23, -0.06) | **0.001** | -0.08 (-0.17, 0.01) | 0.10 |
| Total cholesterol mmol/L | 0.10 (0.05) | 0.07 (0.06) | -0.03 (-0.18, 0.13) | 0.74 | -0.01 (-0.17, 0.16) | 0.96 |
| HDL-c mmol/L | 0.04 (0.01) | 0.12 (0.01)^*^ | 0.09 (0.05, 0.12) | **<0.001** | 0.05 (0.01, 0.09) | **0.022** |
| LDL-c mmol/L | 0.07 (0.05) | 0.07 (0.05) | -0.01 (-0.14, 0.13) | 0.96 | 0.00 (-0.15, 0.14) | 0.98 |
| Body fat mass percentage, % | -1.26 (0.25)^*^ | -2.79 (0.25)^*^ | -1.53 (-2.23, -0.83) | **<0.001** | -0.79 (-1.51, 0.08) | **0.030** |
| Body lean mass percentage, % | 1.22 (0.24)^*^ | 2.66 (0.24)^*^ | 1.44 (0.77, 2.10) | **<0.001** | 0.75 (0.08, 1.43) | **0.029** |
| Visceral fat area, cm^2^ | -3.81 (1.01)^*^ | -9.51 (1.00)^*^ | -5.70 (-8.49, -2.91) | **<0.001** | -2.36 (-5.16, 0.45) | 0.10 |

Estimated means (standard error) of the changes in variables from baseline to month six and between-group differences were derived from the linear mixed-effects model analysis. Model 1 treated group, time, and the group-by-time interaction as the fixed effect factors, and individuals as the random effect factor, while age and sex were controlled as covariates. Model 2 was further adjusted for compliance (compliance score≥3 or compliance score<3) during the 6-month intervention. *P* values for the effects of the interaction between group and time were examined by linear mixed models.

^*^ Statistically significant change from baseline to post-intervention at *p* <0.05.

^a^ Data were log-transformed before analysis due to the skewed distribution.

Abbreviations: HDL-c, high-density lipoprotein cholesterol; LDL-c, low-density lipoprotein cholesterol; MetS, metabolic syndrome; SAA: Smartphone app-based arm (control group); SADA: Smartphone app-based plus dietician arm.

**Table S3 Dietary intake and physical activity levels of participants during the 6-month intervention**

|  | **SAA (N=198)** | **SADA (N=197)** | **Between-group difference**  **(95% CI)** | ***p*-value** |
| --- | --- | --- | --- | --- |
| Total energy intake, kJ | |  |  |  |
| Baseline | 1761±454.3 | 1671.3±451.4 |  | 0.78 |
| Month six | 1659±466.3^*^ | 1543.3±379.4^*^ | -14.8 (-119.4, 89.9) |  |
| Carbohydrate, g^a^ | |  |  |  |
| Baseline | 177.0±34.4 | 179.3±33.3 |  | 0.46 |
| Month six | 160.8±32.3^*^ | 160.6±29.2^*^ | -3.34 (-12.1, 5.42) |  |
| Protein, g^a^ | |  |  |  |
| Baseline | 75.0±18.7 | 76.1±16.1 |  | 0.025 |
| Month six | 76.1±17.9 | 82.3±16.6^*^ | 4.99 (0.65, 9.33) |  |
| Fat, g^a^ | |  |  |  |
| Baseline | 77.7±13.8 | 76.2±14.3 |  | 0.60 |
| Month six | 71.6±13.7^*^ | 69.0±11.9^*^ | -0.96 (-4.56, 2.64) |  |
| Dietary fiber, g/1000 kJ^b^ | |  |  |  |
| Baseline | 0.96 (0.68, 1.22) | 0.98 (0.67, 1.37) |  | 0.97 |
| Month six | 0.89 (0.66, 1.21) | 0.97 (0.69, 1.33) | 0.00 (-0.14, 0.14) |  |
| Physical activity, MET-min/week^b^ | |  |  |  |
| Baseline | 2079 (1272, 3804) | 2337 (1320, 4090) |  | 0.24 |
| Month six | 2385 (1170, 4934) | 2973 (1617, 4745) ^*^ | 0.13 (-0.09, 0.34) ^†^ |  |

Data were observational means ± SD for normally distributed variables and median (interquartile range) for skewed variables. The effects of group, time, and their interaction on variables were examined by linear mixed models (fixed effects = group, time, and group-by-time interaction; random effects = subject), which controlled for age and gender. Post hoc pairwise comparisons were performed. *P* values for the effects of the interaction between group and time were examined by linear mixed models.

^*^ Statistically significant change from baseline to postintervention at *p* < 0.05.

^a^ The macronutrients intakes were adjusted for energy intake using the residual method.

^b^ Data were log-transformed before analysis due to the skewed distribution.

Abbreviations: MET, metabolic equivalent.; SAA, Smartphone app-based arm (control group); SADA, Smartphone app-based plus dietitian arm.

**Table S4 The difference in intervention effects between the SAA and SADA over 6-month intervention in compliant participants ^a^**

|  | **Mean change from baseline (SE)** | | **Between-group difference** | ***p*-value** |
| --- | --- | --- | --- | --- |
|  | **SAA(N=60)** | **SADA(N=122)** |  |  |
| Body weight, kg | -3.60 (0.54)^*^ | -4.73 (0.38)^*^ | -1.13 (-2.42, 0.15) | 0.08 |
| Body weight, % | -4.79 (0.81)^*^ | -6.30 (0.50)^*^ | -1.34 (-2.93, 0.26) | 0.07 |
| Body-mass index, kg/m^2^ | -1.45 (0.20)^*^ | -1.75 (0.14)^*^ | -0.30 (-0.77, 0.17) | 0.21 |
| Body fat mass percentage, % | -2.35 (0.44)^*^ | -3.56 (0.31)^*^ | -1.21 (-2.26, 0.00) | **0.025** |
| Body lean mass percentage % | 2.24 (0.42)^*^ | 3.38 (0.30)^*^ | 1.13 (0.14, 2.14) | **0.026** |
| Visceral fat area, cm^2^ | -8.73 (1.73)^*^ | -13.07 (1.21)^*^ | -4.32 (-8.46, -0.19) | **0.041** |
| Waist circumference, cm | -5.80 (0.66)^*^ | -7.40 (0.46)^*^ | -1.60 (-3.18, -0.01) | **0.048** |
| Systolic blood pressure, mmHg | -2.13(1.25) | -4.23 (0.88)^*^ | -2.10 (-5.10, 0.89) | 0.17 |
| Diastolic blood pressure, mmHg | -1.47 (0.93) | -4.01 (0.65)^*^ | -2.53 (-4.75, -0.31) | **0.026** |
| Fasting glucose, mmol/L | -0.08 (0.06) | -0.06 (0.04) | -0.01 (-0.13, 0.16) | 0.85 |
| Triglycerides mmol/L^b^ | -0.15 (0.05)^*^ | -0.21(0.04)^*^ | -0.06 (-0.19, 0.07) | 0.36 |
| Total cholesterol mmol/L | 0.07 (0.10) | 0.02 (0.07) | -0.04 (-0.28, 0.21) | 0.77 |
| HDL-C mmol/L | 0.12(0.03)^*^ | 0.14(0.02)^*^ | 0.02 (-0.04, 0.08) | 0.52 |
| LDL-C mmol/L | 0.08 (0.09) | 0.04 (0.06) | -0.04 (-0.26, 0.18) | 0.71 |

Estimated means (standard error) of the changes in variables from baseline to month six and between-group differences were derived from the linear mixed-effects model analysis among compliant participants (n=182). Models treated group, time, and the group-by-time interaction as the fixed effect factors, and individuals as the random effect factor, while age and gender at baseline were controlled as covariates. *P* values for the effects of the interaction between group and time were examined by linear mixed models.

^*^Statistically significant change from baseline to post-intervention at *p*<0.05.

^a^ Compliant participants were defined as the participants who attended both baseline and 6-month follow-up with a compliance score≥3.

^b^ Triglyceride data was missing for one participant in the SAA group owing to extreme values (exceeding nine times IQR). Data were log-transformed before analysis due to the skewed distribution.

Abbreviations: HDL-C, high-density lipoprotein cholesterol; LDL-C, low-density lipoprotein cholesterol; SAA: Smartphone app-based arm (control group); SADA: Smartphone app-based plus dietitian arm.

**Table S5** **Mediating effect of the intervention compliance on the between-group difference in BMI change**

|  | **Direct effect (95%CI)** | **Total effect (95%CI)** | **Percentage mediation (95%CI)** | ***p*-value** |
| --- | --- | --- | --- | --- |
| Compliance score | 0.26 (0.08, 0.43) | 0.39 (0.22, 0.57) | 34.3 (19.3, 62.8) | **<0.001** |
| Online courses learning | 0.25 (0.08, 0.42) | 0.26 (0.09, 0.43) | 2.22 (-7.16, 12.3) | 0.56 |
| Weight monitoring | 0.26 (0.10, 0.43) | 0.31 (0.15, 0.49) | 18.4 (3.82, 41.0) | **0.026** |
| Blood pressure monitoring | 0.25 (0.10, 0.40) | 0.25 (0.10, 0.40) | -0.05 (-4.38, 3.54) | 0.95 |
| Smart band using | 0.25 (0.09, 0.42) | 0.25 (0.09, 0.43) | 1.15 (4.44, 8.99) | 0.59 |
| Diet recording | 0.26 (0.09, 0.42) | 0.26 (0.09, 0.42) | 0.03 (-4.38, 3.51) | 0.97 |

The mediation analysis was conducted based on all available data which was tracked weekly during the 6-month intervention period among all randomized participants. The linear mixed models were adopted for the mediation analysis including intervention group, time (by week), and their interaction as fixed effect factors and individual as the random effect factor, adjusting for age, gender, current smoking and drinking, physical activity, educational level, and energy intake at baseline. Intervention compliance refers to the overall compliance score or compliance of each behavior calculated for each week.


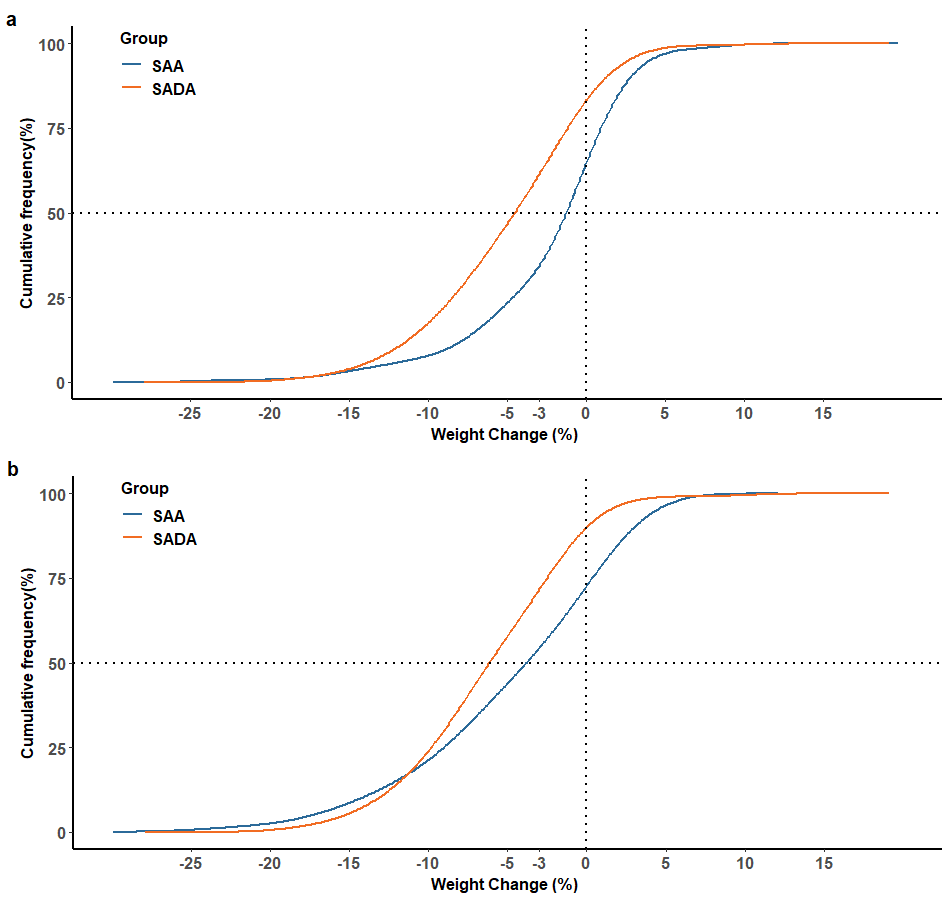


**Fig. S1** Cumulative distribution plot of body weight change from baseline to week 68. Cumulative distribution plot of observed percentage change from baseline in body weight among (**a**) all participants allocated to SAA or SADA (SAA: n=198; SADA: n=197) and (**b**) participants who attended both baseline and 6-month follow-up with a compliance score≥3. (SAA: n = 60; SADA: n = 122). Abbreviations: SAA, Smartphone app-based arm (control group); SADA, Smartphone app-based plus dietitian arm


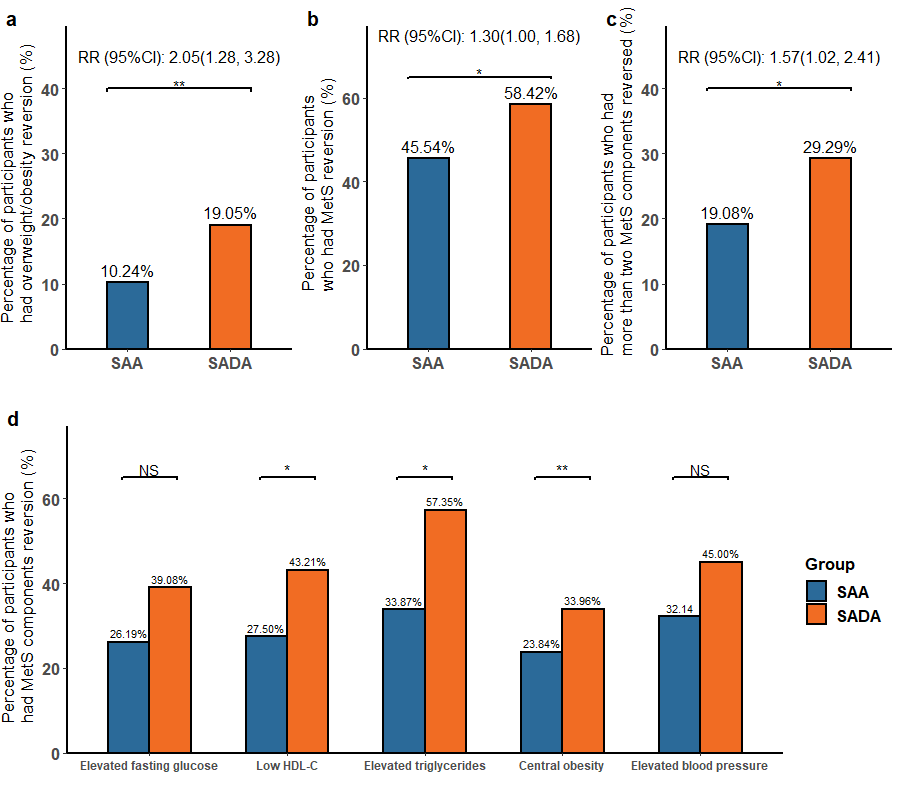


**Fig. S2** Effect of SADA compared to SAA on clinically significant weight change and reversion rates of Mets risk factors over 6-month intervention in per-protocol analysis. **a** Obesity reversion rate at month six among overweight/obesity (BMI≥24 kg/m^2^) participants at baseline (SAA: n = 166; SADA: n = 168). **b** The percentage of participants with more than two Mets components reversed at month six among participants with ≥2 MetS components at baseline (SAA: n = 131; SADA: n = 140). **c** MetS reversion rate at month six among participants with MetS at baseline (SAA: n = 101; SADA: n = 101). **d** Reversion rates of each Mets component at month six among participants with elevated fasting glucose (SAA: n = 84; SADA: n = 87), low HDL-c (SAA: n = 80; SADA: n = 81), elevated triglycerides (SAA: n = 62; SADA: n = 68), central obese (SAA: n = 151; SADA: n = 159), elevated blood pressure (SAA: n = 56; SADA: n = 60) at baseline. ^*^*p*-value<0.05; ^**^*p*-value<0.01. Abbreviations: HDL-c, high-density lipoprotein cholesterol; MetS, metabolic syndrome; SAA, Smartphone app-based arm (control group); SADA, Smartphone app-based plus dietitian arm


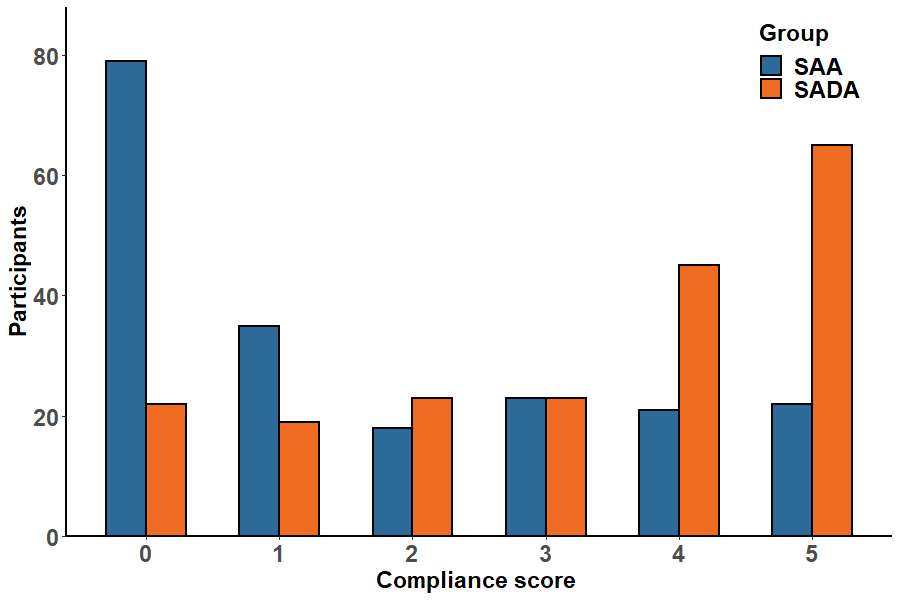


**Fig. S3** The distribution of the overall compliance score in SAA(n=198) and SADA(n=197) over six months. Abbreviations: SAA, Smartphone app-based arm (control group); SADA, Smartphone app-based plus dietitian arm


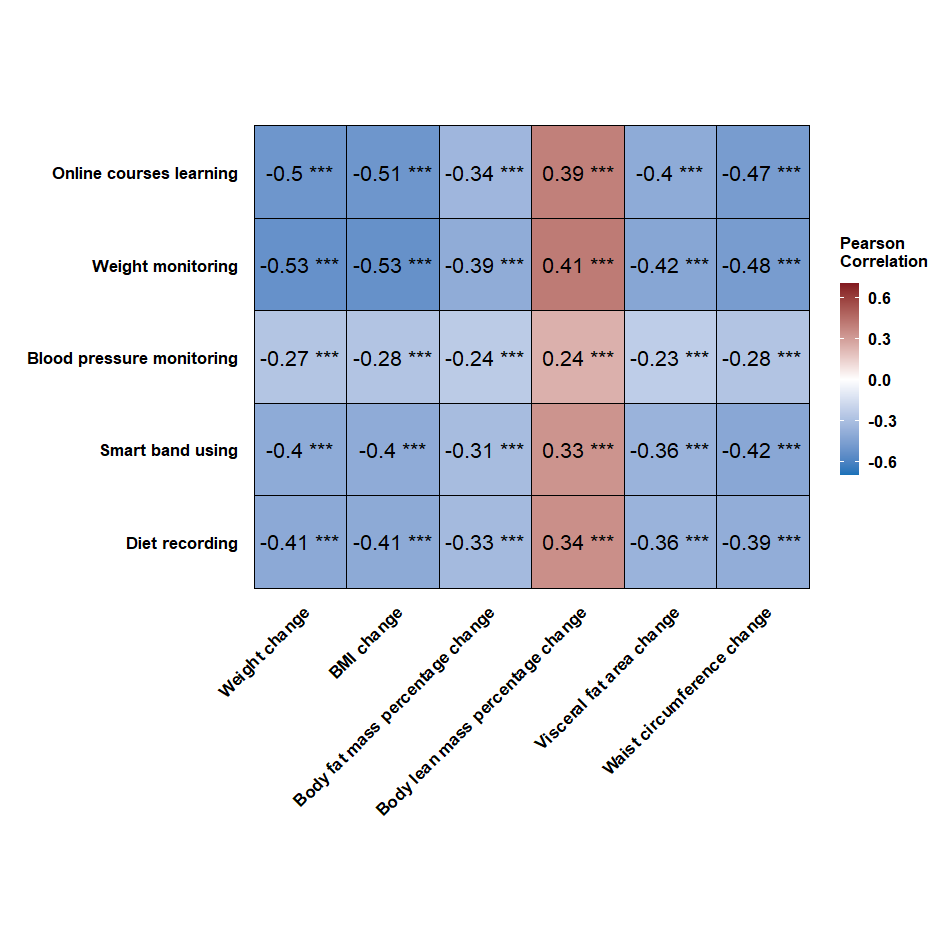


**Fig. S4** Pairwise correlation heatmap of the compliance with five core behaviors and the changes in obesity-related anthropometrics. The pairwise Pearson correlations were conducted among participants who attended the follow-up visit at the end of the 6-month intervention (n=347) with adjustment for age, sex, and intervention group. The colors and the numbers within cells represent Pearson correlation coefficients and the stars refer to the significance. **p*<0.05, ***p*<0.01, ****p*<0.001


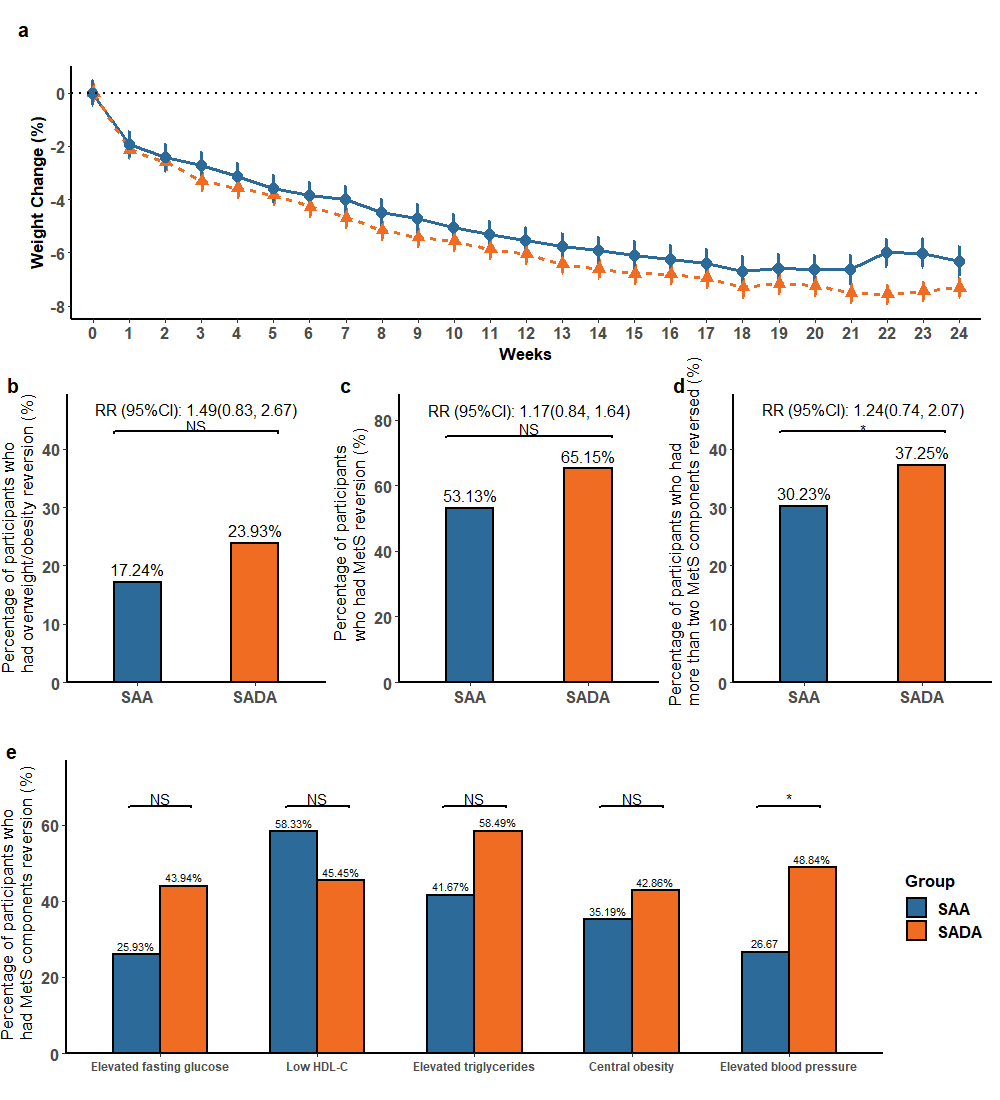


**Fig. S5** Effect of SADA compared to SAA on the trend of weight reduction and reversion rates of overweight, MetS, and MetS components over 6-month intervention among compliant participants. Compliant participants were identified as participants with a compliance score≥3 (SAA: n = 66; SADA: n = 133). **a** The figure shows the mean weight loss (%) monitored by blue-tooth scales over the 6-month intervention period, which were estimated by the linear mixed-effect models, among compliant participants (SAA: n = 66; SADA: n = 133). **b** Obesity reversion rate at month six among overweight/obesity (BMI≥24 kg/m^2^) participants at baseline (SAA: n = 58; SADA: n = 117). **c** MetS reversion rate at month six among participants with MetS at baseline (SAA: n = 32; SADA: n = 66). **d** The percentage of participants with more than two MetS components reversed at month six among participants with ≥2 MetS components at baseline (SAA: n = 43; SADA: n = 102). **e** Reversion rates of each MetS component at month six among participants with elevated fasting glucose (SAA: n = 27; SADA: n = 66), low HDL-C (SAA: n = 24; SADA: n = 55), elevated triglycerides (SAA: n = 24; SADA: n = 53), central obese (SAA: n = 54; SADA: n = 112), elevated blood pressure (SAA: n = 15; SADA: n = 43) at baseline. ^*^*p*-value<0.05; ^**^*p*-value<0.01. Abbreviations: HDL-c, high-density lipoprotein cholesterol; MetS, metabolic syndrome; SAA, Smartphone app-based arm (control group); SADA, Smartphone app-based plus dietitian arm

**Reference**

Alberti KG, Eckel RH, Grundy SM, Zimmet PZ, Cleeman JI, Donato KA, Fruchart JC, James WP, Loria CM, Smith SC, Jr. (2009) Harmonizing the metabolic syndrome: a joint interim statement of the International Diabetes Federation Task Force on Epidemiology and Prevention; National Heart, Lung, and Blood Institute; American Heart Association; World Heart Federation; International Atherosclerosis Society; and International Association for the Study of Obesity. Circulation, 120(16):1640-1645. https://doi.org/10.1161/circulationaha.109.192644

Jones NR, McCormack T, Constanti M, McManus RJ (2020) Diagnosis and management of hypertension in adults: NICE guideline update 2019. Br J Gen Pract, 70(691):90-91. https://doi.org/10.3399/bjgp20X708053

Pan X-F, Wang L, Pan A (2021) Epidemiology and determinants of obesity in China. The Lancet Diabetes & Endocrinology, 9(6):373-392. https://doi.org/10.1016/S2213-8587(21)00045-0
